# Neurobiological Mechanisms Modulating Emotionality, Cognition and Reward-Related Behaviour in High-Fat Diet-Fed Rodents

**DOI:** 10.3390/ijms23147952

**Published:** 2022-07-19

**Authors:** Dorothea Ziemens, Chadi Touma, Virginie Rappeneau

**Affiliations:** 1 Department of Behavioural Biology, University of Osnabrueck, Barbarastrasse 11, 49076 Osnabrueck, Germany; dorothea.ziemens@uni-luebeck.de (D.Z.); ctouma@uni-osnabrueck.de (C.T.); 2 Institute for Experimental and Clinical Pharmacology and Toxicology, Center of Brain, Behavior and Metabolism, University of Luebeck, Ratzeburger Allee 160, 23562 Luebeck, Germany

**Keywords:** rodent, high-fat diet, psychostimulant drugs, energy metabolism, mitochondria, neuroplasticity

## Abstract

Affective and substance-use disorders are associated with overweight and obesity-related complications, which are often due to the overconsumption of palatable food. Both high-fat diets (HFDs) and psychostimulant drugs modulate the neuro-circuitry regulating emotional processing and metabolic functions. However, it is not known how they interact at the behavioural level, and whether they lead to overlapping changes in neurobiological endpoints. In this literature review, we describe the impact of HFDs on emotionality, cognition, and reward-related behaviour in rodents. We also outline the effects of HFD on brain metabolism and plasticity involving mitochondria. Moreover, the possible overlap of the neurobiological mechanisms produced by HFDs and psychostimulants is discussed. Our in-depth analysis of published results revealed that HFDs have a clear impact on behaviour and underlying brain processes, which are largely dependent on the developmental period. However, apart from the studies investigating maternal exposure to HFDs, most of the published results involve only male rodents. Future research should also examine the biological impact of HFDs in female rodents. Further knowledge about the molecular mechanisms linking stress and obesity is a crucial requirement of translational research and using rodent models can significantly advance the important search for risk-related biomarkers and the development of clinical intervention strategies.

## 1. Introduction

The rates of overweight and obesity are rising in children and adults in most parts of the world, representing a major global health challenge [1]. Excessive weight and obesity are partly due to a sedentary lifestyle combined with a high-calorie diet (i.e., a diet rich in sugars and fat, also referred to as a “Western diet”) [2] and can be associated with compulsive eating, binge eating disorders, as well as eating addiction [3,4]. Overweight and obesity increase the risk of developing metabolic diseases (e.g., heart disease, stroke, high blood pressure, diabetes), but also cancer and chronic diseases [5]. Although overweight- and obesity-related metabolic complications can be reduced in a subset of individuals by changes in lifestyle (i.e., balanced diet, healthy eating habits, regular physical exercise), pharmacotherapies and/or surgical procedures, the vast majority of patients exhibit only transient weight loss, which is followed by rebound effects [6,7].

In addition to physical complications, overweight and obesity have a strong impact on mental health [8,9]. In particular, they increase the risk for developing depressive and anxiety disorders as well as substance-use disorders (SUD) (e.g., illegal drugs, nicotine or alcohol) [10,11,12,13,14]. The latter disorders have a high prevalence in the population worldwide and result in a high degree of comorbidity themselves [15,16,17].

In recent years, there has been increasing interest in understanding the shared neurobiological substrates of obesity and SUD [18]. In both disorders, the saliency of a specific type of reward (food or drug) becomes exaggerated, leading to overeating or compulsive drug abuse, respectively [19]. Food and drug reward activate overlapping brain nuclei [20,21,22], which complicates research designed to understand the neurobiological underpinnings of behaviours motivated by rewards in obesity and SUD [23,24].

A theoretical mechanism that has dominated the field for many years is the dysregulation of the brain reward pathways involving the dopamine (DA) system [25], which responds to drugs of abuse and is critical in the development of SUD [26,27]. The consumption of palatable food leads to the activation of the DA reward circuitry [28]. Additionally, limiting the intake of rodent chow diet increased the sensitivity to cocaine and amphetamine, two psychostimulant drugs widely used and abused that increase dopaminergic transmission [29,30,31]. Furthermore, limiting the access to a high-calorie diet produced dopaminergic adaptations similar to those observed in responses to rewarding stimuli in the nucleus accumbens (NAc), a brain area involved in reward processing and drug addiction [32,33].

In brain imaging studies, obese patients present a downregulation of striatal DA function [34], with individuals with the largest body mass index having the lowest dopamine receptor 2 (D2R) availabilities [34,35]. The reduction in D2R availability was associated with the reduced metabolism of various prefrontal brain regions [35]. Because variations in striatal D2R availability have been linked to changes in the reinforcing value of both food and drugs of abuse [34,36], it has been proposed that obese patients and drug addicts may share neuroadaptations in dopaminergic pathways. These neuroadaptations may regulate neuronal systems associated not only with reward and motivation, but also with inhibitory control, salience attribution, and emotional reactivity. Furthermore, obese patients exhibited greater activation of brain regions involved in reward and attention in response to palatable food images or consumption compared to normal-weight subjects [37,38]. Thus, these neuroadaptations may produce a reduced state of reward, which is then possibly compensated for by the overconsumption of palatable food and/or the abuse of drugs [39].

Multiple intertwined genetic, psychosocial, and neuro-immuno-endocrine factors have been proposed to contribute to the association of obesity and SUD, including the gut–brain axis, inflammation and oxidative stress [40,41,42,43,44]. In particular, much evidence shows that overweight and obesity are associated with alterations in oxidative stress and mitochondrial functions in peripheral organs and in the brain [45,46,47,48]. In parallel, drugs of abuse have been shown to increase oxidative stress occurring in dopaminergic neurotransmission [49,50,51,52,53]. A better knowledge of the precise neurobiological mechanisms contributing to the comorbidity of obesity and SUD, and their independent association with depressive and anxiety disorders, is needed. This could help understand how palatable food and drugs of abuse disrupt the reward circuit and lead to changes in compulsive and affective behaviours. This may also inform novel therapeutic targets and preventive efforts for obesity and SUD.

## 2. Aim of the Present Review

In this context, the aims of this narrative review are the following.

First, we recapitulate some of the predominant behavioural changes caused by exposure to palatable food and in combination with psychostimulant drugs (i.e., cocaine and amphetamine) in juvenile, adolescent, and adult rodents. Because of the high prevalence of obesity in pregnant women [54] and the short- and long-term negative consequences of poor nutrition during pregnancy and lactation on both the mother and child [55], we also examine the impact of palatable food in dams and their offspring.

Second, we describe the neurobiological mechanisms that may contribute to disruptions in behaviour and metabolic functions by palatable food in rodents. At the molecular level, we focus on brain energetics, mitochondrial function, oxidative stress, neuroplasticity and neuro-inflammation. A more detailed analysis of the molecular mechanisms involving the microbiota–gut–brain axis falls outside the scope of the current review (for more information see [56,57,58,59] and discussion Section 7.2.2).

Finally, we attempt to address the issue of whether the consumption of high-energy diets and psychostimulant drugs leads to overlapping changes in brain metabolism and plasticity that alter emotional states and thus modulate subsequent behaviours.

## 3. Important Considerations on the Use of High-Fat Diet Treatments in Rodents

Over the past century, diet-induced obesity models have been used in rodents to investigate the body and brain alterations that occur during the progression of obesity and in the presence or in the absence of predetermined genetic alterations [60]. They include various approaches (i.e., diets rich in fat and/or sugar, genetic models, pharmacological models) and produce, in various degrees, alterations in energy metabolism resembling those found in obese patients. For example, high-energy diets markedly increase body weight and adiposity, and induce a pre-diabetic phenotype (e.g., hyperglycaemia, hyperinsulinemia, insulin resistance, glucose intolerance). Moreover, they produce hormonal dysregulation, hypothalamic neuropeptidergic adaptations and low-grade inflammation [61,62,63,64,65].

For the behavioural level, the current literature is filled with examples in which long-term exposure to high-energy diets alters cognitive performance and modulates emotionality [66,67,68,69,70,71,72]. The latter can be defined as the measure of emotional behaviour related to undirected escape, avoidance of specific stimulus or area, immobility and sympathetic nervous system activation (e.g., heart rate, urination, defecation) [73]. However, there are numerous discrepant findings, for instance in rodent models of depression [69]. The main theoretical premise behind the inconsistencies concerns variations in experimental designs (e.g., diet content and duration, exposure period; sex, age, species and genetic background of the animals used; behavioural tests).

Recent criticism of the preclinical (mainly rodent) literature indicates that the commonly used commercial high-energy diets may not necessarily model the dietary habits associated with overweight and obesity in humans [74]. For instance, rodent high-fat diets (HFDs) typically contain either ≈45% or 60% kcal from fat and are characterised by varying fatty acid composition [62,74]. Because the currently recommended total fat intake for adult humans ranges from 20 to 30% [75,76] and the average European/American diets contain approximately 28.5 to 46.2% kcal from fat [74,77,78], the use of rodent HFD containing ≈45% kcal from fat seems the most appropriate diet from a translational point of view.

Here, we aimed to reduce the heterogeneity of findings between studies and parallel the fat content found in the average European/American diets. Thus, we compare the behavioural effects of HFD treatments containing ≈25–50% kcal from fat (predominantly 45%) with control (CON) diets providing 3–18% fat (usually 10%). Studies that used a rodent HFD containing a very high amount of fat (≈60% kcal), which rapidly produce extreme obesity [62] and increase pup cannibalism in dams [79], were excluded from the present review.

With the exception of the studies on maternal HFD and a few other studies, all reviewed studies used male mice or rats. To assess biological parameters indicative of metabolic dysfunction, the negative impact of the HFD treatment on energy metabolism was almost systematically verified (e.g., body weight gain, increased calorie intake, hyperglycaemia, hyperinsulinemia, dyslipidaemia, peripheral inflammation) (see details in Supplementary Table S1).

## 4. Impact of a High-Fat Diet Treatment on Emotionality, Cognition and Reward-Related Behaviour

The impact of HFD treatment on rodent behaviour is summarized in Table 1.

### 4.1. Impact of a Maternal High-Fat Diet Treatment in Dams

HFD consumption during pre- and post-partum periods, two metabolically challenging periods, produced mixed changes in the gestational body weight, but generally increased adiposity and induced hyperleptinemia in dams (see Supplementary Table S1). The behavioural profile of the dams showed that maternal HFD had a negative impact on maternal behaviour, especially regarding nest building and pup licking/grooming [80,81].

Interestingly, one study showed that maternal HFD had no significant impact on nursing behaviour in breeding dams. However a reduction in the quality of maternal care was observed in dams from generation F3, which were selectively bred for diet-induced obesity or diet-induced resistance [82].

So far, few studies have investigated the link between obesity-related factors and increased risk for maternal diseases as well as the importance of inherited predisposition to HFD-induced obesity. Further work is therefore needed to understand how obesity during pregnancy and lactation influence the maternal metabolic and behavioural adaptations necessary to bring about new life.

### 4.2. Impact of a Maternal High-Fat Diet Treatment in Offsspring

To our knowledge, very few studies examined the impact of HFD treatment (containing ≈ 25–45% kcal from fat) applied to breeding dams during pregnancy and lactation on the behavioural and metabolic phenotypes of the offspring (see Table 1, Supplementary Table S1).

The offspring of mothers fed a HFD during pregnancy showed impaired learning in an operant conditioning paradigm when tested in adulthood [83]. This was indicated by an increased number of sessions required to press an operant lever and obtain a reward [83]. However, mice from HFD-fed dams and CON-fed dams did not significantly differ regarding their motivation to obtain a reward and their appetitive and/or consummatory drive (i.e., a similar number of rewards was received) [83]. They also did not significantly differ regarding locomotion and anxiety-related behaviour [83]. Because of the very limited data available regarding the long-term behavioural consequences of a HFD during prenatal development, further work is required to draw any conclusion.

A few studies investigated the effects of a HFD during the pre- and post-partum periods on the offspring’s development, behaviour and cognition. Juvenile offspring of mothers fed a HFD during pregnancy and lactation showed an increased preference for a 1% corn oil solution [84], changes in voluntary running wheel activity [85] and increased passive stress-coping behaviour [86]. No significant differences between the offspring of HFD-fed dam and CON-fed dams were seen in locomotion, anhedonia and learning and memory [86,87]. Regarding psychostimulant-related behaviour, the offspring displayed reduced amphetamine-induced locomotion and sensitization [87].

Compared to offspring of CON-fed mothers during pregnancy and lactation, adolescent offspring of mothers fed a HFD treatment during pregnancy and lactation showed no significant differences in locomotion and the rewarding or motivational effects of cocaine in the cocaine self-administration paradigm [88]. When tested in adulthood, a few studies failed to also detect an effect on a maternal HFD treatment on behavioural phenotypes, notably locomotion, anxiety-related behaviour and cognitive performance [83,89].

It is important to mention that the maternal HFD produced long-term changes in the reactivity of the hypothalamic–pituitary–adrenal axis, the core mediator of neuroendocrine stress responses [90]. In addition, cognitive deficits could be identified by a post-weaning exposure to HFD treatment [89], suggesting that a second hit stressor may be necessary to unmask programmed behavioural defects of the early nutritional environment.

### 4.3. Impact of a High-Fat Diet Treatment during the Onset of Puberty and Early Adolescence

#### 4.3.1. Cognition and Emotionality

The consumption of a HFD during the onset of puberty and early adolescence period (here referred to as “juvenile HFD”, involving 4–6-week-old mice and 3–9-week-old rats [91] produced long-term negative consequences on hippocampus-dependent cognition (see Table 1). This was observed in several tasks assessing spatial memory, recognition memory and fear-aggravated memory [92,93,94,95,96]. Similar to the effects of a metabolic challenge (i.e., exposure to HFD), chronic exposure to physical and/or psychosocial stressors early in life produced long-term impairments in learning and memory in rodents, specifically for the spatial domain [97,98]. However, the biological and signalling pathways leading to alterations in cognitive performance for the different types of stress may differ.

Regarding emotionality, a juvenile HFD treatment did not reliably increase anxiety-related behaviour in various paradigms [92,93,95,99,100,101,102,103,104]. It also had no major impact on locomotion and stress-coping style in the forced swim test [92,93,94,95,100,101,102,103,105,106] (see Table 1).

#### 4.3.2. Psychostimulant-Related Behaviour

Concerning psychostimulant-related behaviour (see Table 1), the outcomes of a juvenile HFD treatment were dependent on its schedule of availability. When given ad libitum, a juvenile HFD treatment weakened cocaine- and amphetamine-related memories in the conditioned place preference (CPP) paradigm, especially for the lower drug doses administered [99,107,108,109]. When time-restricted access was provided, juvenile HFD treatment leading to binge eating enhanced the acquisition and reinstatement of cocaine self-administration [107]. The results of these studies suggest that palatable food can modulate psychostimulant-rewarding and reinforcing properties under certain patterns of food administration. In particular, they highlight that animals that develop a pattern of binge eating on palatable food are more vulnerable to psychostimulant drugs (referred to as cross-sensitization) [111]. The neurobiological mechanisms by which HFD treatments and psychostimulant drugs act to promote compulsive behaviour warrant further investigation.

One crucial factor to consider is the importance of the social housing conditions of the animals. In fact, a juvenile intermittent HFD treatment led to binge eating in single-housed mice and reduced their cocaine-induced CPP, but increased cocaine-induced CPP in group-housed animals that did not show binge-eating behaviour [112]. This study suggests that palatable food may work as an alternative reward to psychostimulant drugs and stimulates the same brain pathways as psychostimulants do. However, the mechanisms by which social housing conditions modulate the rewarding properties of HFD treatment are presently unclear.

Regarding the behaviour of animals during the exposure to psychostimulant drugs (see Table 1), a juvenile HFD treatment was found to modulate the behavioural sensitization to amphetamine [108,113] and had a minor impact on cocaine-induced anxiolytic and “antidepressant” effects [106,107]. During cocaine withdrawal, HFD treatment facilitated the extinction of cocaine-induced CPP, increased cocaine-induced locomotion, and produced anxiolytic and antidepressant effects [99,106]. Interestingly, preventing access to the HFD treatment increased anxiety-related behaviour, cocaine-induced locomotion and cocaine-induced CPP for a subthreshold cocaine dose [107], suggesting that withdrawal from HFD could potentiate psychostimulant-rewarding properties.

The long-term consequences of chronic consumption of (and withdrawal from) palatable foods during the early adolescence period remain unclear but might lead to alterations of the brain reward system that have been associated with obesity and metabolic disorders.

### 4.4. Impact of a High-Fat Diet Treatment during Late Adolescence and Young Adulthood

Turning to the experimental evidence on the impact of HFD treatment during late adolescence and young adulthood (i.e., in 6–9-week-old mice and 9–10-week-old rats [91]), (see Table 1), the summarized data show that an adolescent HFD treatment increased anxiety-related and anhedonia-like responses, without reliably affecting stress-coping style [114,115,116,117,118]. It also reduced locomotion, exploration but not social behaviours [116,117,119,120]. In contrast to HFD treatment in juveniles, HFD treatment in adolescents had no major impact on hippocampus-dependent learning and memory [116,121,122]. Similar results of increased emotionality and reduced reward responsiveness have been reported in rodents exposed to physical and/or psychosocial stressors during adolescence [123,124].

### 4.5. Impact of a High-Fat Diet Treatment in Adulhood

HFD treatment in adulthood (beyond the age of 9 weeks in mice and 10 weeks in rats) reduced the hedonic response in the sucrose preference test [125], but it did not produce major behavioural alterations concerning anxiety, locomotion/exploration, and learning and memory [126,127]. However, with such a small number of studies, caution must be applied, as these findings might not reflect the overall impact of an adult HFD treatment on emotionality, cognition and reward-related behaviour in rodents.

HFD treatment in adults altered cocaine-induced locomotor and antidepressant effects [128], and increased amphetamine-induced locomotion, but without inducing a behavioural sensitization to amphetamine [129]. Interestingly, withdrawal from amphetamine significantly increased body weight gain and produced hyperphagia in HFD-fed animals, suggesting that HFD consumption may alleviate reward deficits mediated by psychostimulant withdrawal. Because the increase in food consumption in amphetamine-withdrawn rats was specific to the laboratory chow rather than the HFD [129], further work is needed to determine how psychostimulant drugs and HFD interact to produce long-lasting sensitization of reward-related behaviours.

### 4.6. Conclusions

In light of the reported findings, it is conceivable that the strong effects of HFD treatment on cognitive performance and reward-related behaviour were seen in both juvenile and adolescent rodents. Indeed, during the pre-pubertal phase and adolescence, rodents undergo dramatic hormonal, neurobiological and behavioural changes [130,131,132]. In particular, the limbic system (notably the hippocampus and amygdala) as well as cortical regions involved in emotional and learning processing undergo structural and functional maturation [133]. Data from several studies have identified these periods as critical time windows during which stress exposure produces short- and long-term effects on emotionality and cognition [134,135,136,137], as well as associated neural structure and function [138,139,140]. In humans, adolescence is considered a window of vulnerability to pathological development [141,142]. Adolescents are particularly sensitive to reward and often increase their consumption of palatable foods, such as a high-fat diet [143], which could lead to obesity.

Because pre-puberty and adolescence are critical periods of neurobehavioural reorganization necessary for life-long cognitive processes and reward processes, the possible mechanisms underlying the marked vulnerability to the detrimental effects of HFD treatment may involve changes in energy metabolism and neuronal plasticity. Such mechanisms are discussed in the next section.

## 5. Impact of a High-Fat Diet Treatment on Molecular Correlates of Energy Metabolism and Plasticity

As reviewed above, poor dietary choices during crucial stages of development can have a detrimental impact on emotionality, cognition and reward-related behaviours. Therefore, what are the effects of HFDs on underlying brain mechanisms?

The impact of HFD treatment on energy metabolism, molecular correlation of mitochondrial dysfunction (e.g., redox imbalance, oxidative damage and inflammation) and neuroplasticity in rodents is summarized in Table 2. Data are sorted by brain area and developmental period (see details in Supplementary Table S1).

### 5.1. Impact of a High-Fat Diet Treatment on the Cerebral Cortex

#### 5.1.1. Energy Metabolism

Regardless of the time at which it was applied, HFD treatment had a minor impact on glucose, amino acid and phospholipid metabolism in the cortex [105,119,127]. However, it seemed to have induced a slight molecular insulin resistance, as indicated by increased insulin level and a lack of activation response of key players of the insulin/phosphatidylinositol 3-kinase (PI3K)/Akt/glycogen synthase kinase-3β (GSK-3β) cascade [119].

#### 5.1.2. Mitochondria-Related Functions and Oxidative Stress

The data gathered show that HFD treatment in both juveniles and adults had a minor impact on cortical mitochondrial bioenergetics (i.e., expression of mitochondrial respiratory chain complexes, respiratory spare capacity, ATP production) [105,144] and the cell redox state [121,127,144]. One study in HFD-fed juvenile animals found a significant decrease in the level of superoxide dismutase (SOD), the first line of defence against oxidative stress, and the anti-oxidant glutathione (GSH) [144]. This was associated with enhanced lipid peroxidation, indicated by increased level of malondialdehyde, and modest neuro-inflammation shown by the variations in the expression of some cytokines (e.g., tumour necrosis factor alpha and interleukins 1β) [144]. Two other studies in HFD-fed juvenile or adolescent animals observed no significant changes in the level of GSH and hydroxynonenal (HNE), another indicator of lipid oxidation [121,127]. No significant changes were seen in the expression of glial fibrillary acidic protein (GFAP) and IBA1 (allograft inflammatory factor 1), a marker of activated microglia [127].

Interestingly, alterations in the redox balance were found in cortical synaptic mitochondria from HFD-fed animals, which also showed alterations in several mitochondrial respiration parameters (i.e., reduced basal respiration, ATP production, proton leak and increased degree of coupling) [144]. Thus, it is possible that HFD treatment produced detrimental effects particularly localized at the synapses.

#### 5.1.3. Neuroplasticity and Neuroinflammation

Because synaptic mitochondria regulate synaptic transmission, variations in synaptic mitochondrial respiration accompanied by increased oxidative stress would be expected to be associated with changes in neurotransmission and neuroplasticity. However, HFD treatment had only a minor impact on synaptic plasticity in the cortex [115,121,127,144]. As for maternal HFD treatment, it had no significant impact on dopaminergic markers in the prefrontal cortex of juvenile offspring [87]. It is noteworthy that HFD treatment in adolescents modulated the expression of brain-derived neurotrophic factor (BDNF), a major regulator of synaptic transmission and plasticity [144]. Additionally, it reduced the synaptic BDNF protein synthesis and the phosphorylation of BDNF upstream factor cyclic AMP-responsive element-binding protein (CREB) in cortical synaptosomal fractions [144], again highlighting the marked effects of HFD treatment on BDNF-related molecular plasticity at the synaptic level.

### 5.2. Impact of a High-Fat Diet Treatment on the Hippocampus

#### 5.2.1. Energy Metabolism

In the hippocampus, a critical brain area for learning and memory, HFD treatment had a minor influence on glucose metabolism and insulin signalling, and no significant impact on glucose transport, amino acid and phospholipid metabolism [105,119,122,127]. As observed for the cerebral cortex, an HFD in adolescence might be associated with a minor molecular insulin resistance, as shown by the lack of activation response of Akt [119]. It might also be associated with reduced glycolysis processes, as suggested by the significant increase in the expression of glucose-6-phosphate and the reduced activity of the pyruvate dehydrogenase enzyme [105].

#### 5.2.2. Mitochondria-Related Functions and Oxidative Stress

The available evidence shows no major impact of HFD treatment in juveniles and adults on oxidative capacity and redox balance of the hippocampus. Indeed, mixed changes were seen in the protein expression of some respiratory chain complexes and no significant impact was found on citrate synthase activity and ATP production or anti-oxidant level [94,121,127]. It also revealed contrasting signs of lipid peroxidation, astrogliosis, microgliosis and discrepant changes in cytokines release [94,103,117,121,126,127].

#### 5.2.3. Neuroplasticity and Neuroinflammation

In contrast to observations made in the cortex, HFD treatment had a major impact on hippocampal neurotransmission and synaptic plasticity in all periods examined, but especially during adolescence. For example, HFD treatment significantly affected serotonergic transmission [117,118]. HFD treatment reduced the expression of BDNF, as well as that of key synaptic proteins involved in vesicle trafficking and receptor anchoring; it also reduced the number of dendritic spines and produced some changes in the cyto-architecture of the hippocampus [117,121,122,127]. Similarly, maternal HFD treatment decreased the length of dendritic spines (without affecting the spine density) and altered astrocyte morphology [89,145].

### 5.3. Impact of a High-Fat Diet Treatment on the Hypothalamus

#### 5.3.1. Energy Metabolism

Unfortunately, studies that examined the impact of HFD treatment on the hypothalamus during adolescence are lacking. Regarding HFD treatment in juveniles and adults, a close look at the data indicates that it did not affect hypothalamic glucose, amino acid or phospholipid metabolism [127,146,147]. Nonetheless, it potentially produced alterations in insulin signalling, as indicated by increased insulin receptor expression in the arcuate nucleus (ARC), an area of particular importance to energy homeostasis [146,147].

**Table 2:** Literature overview of the impact of a high-fat diet treatment on molecular correlates of energy metabolism and plasticity in the brain of rodents. Description: Studies are organized by period in which the high-fat diet (HFD) treatment was applied (i.e., before puberty, during adolescence or in adulthood). For the effects of a maternal HFD treatment, studies are organised by the developmental period (i.e., before puberty or early adolescence, during late adolescence or in adulthood) in which the offspring were tested. The numbered references refer to the bibliography section.

Brain Area & Ref.	Overall Function	Outcomes		
		⬆ (Increased)	⬇ (Decreased)	⬌ (Not Changed)
**Maternal HFD treatment** **Testing in offspring**				
**PFC** [87]	**Neuronal func.**			Level of DA, DOPAC
**HC** [89,145]	**Neuronal func.**		Dendritic spine length	Dendritic spine density DCX-positive cells
	**Neuroinflam.**	Astrocyte process number and total length		
**AMY** [145]	**Neuronal func.**		Dendritic spine length	Dendritic spine density
**NAc** [84,87]	**Neuronal func.**	Level of DA, DOPAC *Ddr2* mRNA *Htr1a* mRNA		TH density fibres Expression of TH, DAT, D1/2R
**STR** [85,87]	**Neuronal func.**			Expression of TH, DAT, D1/2R
**VTA** [84,87]	**Neuronal func.**	*Htr1a* mRNA	TH positive neurons § *Th* mRNA §	TH positive neurons § *Th* mRNA §
**Juvenile HFD treatment**				
**CC** [105]	**Glucose metab.**			Glucose transport, glycolysis
	**Insulin signaling**	Insulin level		Insulin sensitivity
	**OXPHOS**	Expression of ETC CIV-V	Spare respiratory capacity	Expression of ETC CI-III, basal OCR, State 3 OCR, level of ATP
**HC** [103,104,105]	**Glucose metab.**		Glycolysis	Glucose transport
	**Insulin signaling**			Insulin level and sensitivity
	**OXPHOS**		Expression of ETC CI-II	Expression of ETC CI-V, level of ATP
	**MT biogenesis**			Expression of PGC1α, PPARγ
	**Oxidative stress**	Lipid peroxidation		Anti-oxidant defenses (SOD, GPX)
	**Neuronal func.**		Dendritic spines, expression of SYP	Expression of BDNF
	**Neuroinflam.**	Level of IL10, micro/astrogliosis	Level of IL6	Level of IL6, *IL1β*, TNFα, microgliosis
**HYP** [104,147,148]	**Insulin signaling**	*Ins* mRNA		*Mtor*, *Irs1* mRNA
	**OXPHOS**			Expression of ETC CI-V, level of ATP
	**Oxidative stress**			Anti-oxidant defenses (SOD, CAT), lipid peroxidation
	**Neuroinflam.**	Microgliosis		*Tgfβ* mRNA, microgliosis
**AMY** [103,104]	**Neuroinflam.**	Level of TNFα, microgliosis		Level of IL6
**NAc** [107,108,113]	**Insulin signaling**	*Mtor* mRNA		
	**Neuronal func.**	Expression of D1R	Expression of DAT	Basal DA release; level of DA, DOPAC; expression of TH, DAT, D1/2R
**VTA** [113]	**Neuronal func.**			Spontaneous/bursting DA activity Expression of TH, DAT
	**HFD treatment in late adolescence**			
**CC** [115,117,119,121]	**Insulin signaling**		Insulin sensitivity	
	**Oxidative stress**			Anti-oxidant defenses (GSH, GSSG), lipid peroxidation
	**Neuronal func.**	*Ddr2* mRNA	*Gabbr1/2* mRNA	Expression of BDNF, SYP *Drd1* mRNA
	**Neuroinflam.**		*Il1β*, *Il2* mRNA	*Il10*, *Il4*, *Il6*, *Tnfα*, *Tgfβ*, *Ifnγ* mRNA
**HC** [117,118,119,121]	**Insulin signaling**		Insulin sensitivity	
	**Oxidative stress**			Anti-oxidant defenses (GSH, GSSG), lipid peroxidation
	**Neuronal func.**		Nissl staining, *Bdnf* mRNA, level of 5-HT; *Htr1a*, *Slc6a4*, *Ido2* mRNA	CA1 pyramidal layer thickness, CA1 LTP, expression of PSD95, BDNF, SYP
	**Neuroinflam.**	*Il1β*, *Il2*, *Il6*, *Il17* mRNA	*Il10* mRNA	*Il4*, *Tnfα*, *Tgfβ*, *Ifnγ* mRNA
**HYP** [149]	**Neuroinflam.**			*IL1β* mRNA, astrogliosis
**Adult HFD treatment**				
**CC** [127,144]	**Glucose metab.**			Level of glucose, lactate
	**AA/PL metab.**		Level of PEA	Level of Gln, Glu, GABA
	**OXPHOS**		State 3 OCR, Spare respiratory capacity	
	**Oxidative stress**	Lipid peroxidation	Anti-oxidant defenses (GSH, GSSG, SOD)	Anti-oxidant defenses (GSH, As)
	**Neuronal func.**		Expression of BDNF	Level of NAA, expression of PSD95, SYP, SYN, VGLUT1/2, VGAT
	**Neuroinflam.**	Level of TNFα, *IL1β*		Micro/astrogliosis
**HC** [126,127]	**Glucose metab.**			Level of glucose, lactate
	**AA/PL metab.**			Level of Gln, Glu, GABA, PEA
	**Oxidative stress**			Anti-oxidant defenses (GSH, As)
	**Neuronal func.**		Expression of SYN, VGLUT1, VGAT	Level of NAA, expression of PSD95, SYP, VGLUT2
	**Neuroinflam.**			*Tnfα*, mRNA, microgliosis
**HYP** [127,146]	**Glucose metab.**			Level of glucose, lactate
	**Insulin signaling**	*Ins* mRNA		
	**AA/PL metab.**			Level of Gln, Glu, GABA, PEA
	**Oxidative stress**			Anti-oxidant defenses (GSH, As)
	**Neuronal func.**		Expression of VGLUT1/2, VGAT	Level of NAA, expression of PSD95, SYP, SYN
	**Neuroinflam.**	Level of TNFα, *IL1β*, IL6, astrogliosis		Microgliosis

Abbreviations: 5-HT: serotonin; AA: amino-acid; AMY: amygdala; As: ascorbate; ATP: adenosine triphosphate; BDNF/*Bdnf*: protein/gene coding for brain-derived neurotrophic factor; CA1: region 1 of the cornu ammonis; CAT: catalase; CC: cerebral cortex; D1R/*Drd1*: protein/gene coding for dopamine receptor 1; D2R/*Drd2*: protein/gene coding for dopamine receptor 2; DA: dopamine; DAT: dopamine transporter; DCX: doublecortin; DOPAC: 3,4-dihydroxyphenylacetic acid; ETC CI-V: electron transport chain complex I-V; GABA: gamma-aminobutyric acid; *Gabbr1/2*: gene coding for gamma-aminobutyric acid type B receptor subunit 1 or 2; Gln: glutamine; Glu: glutamate; GPX: glutathione peroxidase; GSH: glutathione; GSSG: oxidised glutathione; HC: hippocampus; HFD: high-fat diet; *Htr1a*: gene coding for 5-hydroxytryptamine receptor 1A; HYP: hypothalamus; *Ido2*: gene coding for indoleamine 2,3-dioxygenase 2; *Ifnγ*: gene coding for interferon; IL/*Il*: protein/gene coding for interleukin; *Ins*: gene coding for insulin; *Irs1*: gene coding for insulin receptor substrate 1; LTP: long-term potentiation; metab.: metabolism; MT: mitochondria; *Mtor*: gene coding for mechanistic target of rapamycin kinase; NAA: N-acetyl aspartate; NAc: nucleus accumbens; Neuroinflam: neuro-inflammation; Neuronal func.: neuronal function; OCR: oxygen consumption rate; OXPHOS: oxidative phosphorylation; PEA: palmitoylethanolamide; PFC: prefrontal cortex; PL: phospholipid; PGC1α: peroxisome proliferator-activated receptor gamma coactivator 1-alpha; PPARγ: peroxisome proliferator-activated receptor gamma; PSD95: postsynaptic density protein 95; *Slc6a4*: gene coding for sodium-dependent serotonin transporter; SOD: superoxide dismutase; STR: striatum; SYN: syntaxin; SYP: synaptophysin; *Tgfβ*: gene coding for transforming growth factor beta; TH/*Th*: protein/gene coding for tyrosine hydroxylase; TNFα/*Tnfα*: protein/gene coding for tumour necrosis factor; VGAT: vesicular GABA transporter; VGLUT1/2: vesicular glutamate transporter 1 or 2; VTA: ventral tegmental area. Symbols: §: outcomes differing based on the age of animals at the time of testing.

#### 5.3.2. Mitochondria-Related Functions and Oxidative Stress

HFD treatment in juveniles had no significant impact on hypothalamic mitochondrial respiration and energy storage during oxidative phosphorylation. This was shown by the absence of significant changes in the activity of respiratory chain complexes and ATP production [147,148]. It also had no significant impact on the cell redox state [147,148]. For instance, it did not alter the expression of first-line antioxidant defences, SOD, catalase (CAT) and GSH [147]. Additionally, it did not alter the total anti-oxidant status and did not increase oxidative damage to lipids [147,148].

HFD treatment in juveniles produced slight alterations in hypothalamic mitochondrial morphology [147,150]. A decreased mitochondrial surface area was reported in the paraventricular nucleus of the hypothalamus (PVN), although no significant changes were seen in the mitochondria type, aspect ratio and surface density coverage [147]. Thaler and colleagues (2012) found divergent mitochondria ultrastructure in pro-opiomelanocortin (POMC) neurons of the ARC. In this study, the morphological examination of mitochondria displayed homogenous, compact electron-dense lumens with well-organised, parallel-oriented cristae in mitochondria in chow-fed rats but not in HFD-treated rats [150].

#### 5.3.3. Neuroplasticity and Neuroinflammation

In the hypothalamus, HFD treatment in adults was found to produce astrogliosis as well as reduce the protein expression of vesicular glutamate transporter and GABA transporters, possibly reducing the availability of excitatory and inhibitory vesicles for neurotransmission [127,149]. However, no significant impact of HFD treatment was seen on the expression of synaptic proteins, neuronal integrity and microgliosis [104,127,147].

### 5.4. Impact of a High-Fat Diet Treatment on the VTA-NAc DA System

#### 5.4.1. Impact of a High-Fat Diet Treatment

To our surprise, there has been little research on the impact of HFD treatment on brain metabolism and plasticity within brain regions that are potentially involved in the behavioural overlap of consumption of HFD and psychostimulant-related behaviour.

For example, maternal HFD treatment had a significant impact on the basal functionality of the ventral tegmental area (VTA)–nucleus accumbens (NAc) DA pathway, which plays a critical role in reward-relevant behaviours and emotional behaviours, especially following stress exposure [151]. For example, juvenile offspring from HFD-fed dams showed a significant increase in the level of the DA neurotransmitter and in 3,4-Dihydroxyphenylacetic acid (DOPAC, a metabolite of DA) in the NAc [87]. No significant changes were seen in the expression of the DA transporter (DAT), DA receptors and tyrosine hydroxylase (TH), the rate-limiting step in this synthesis of DA [87]. In the VTA, juveniles from HFD-fed dams exhibited changes in the expression of TH and the number of TH-positive neurons [87]. Further studies are therefore needed to determine how HFD treatment produces neuro-adaptations in the mesolimbic DA system during the pre- and the post-partum periods to influence reward-related behaviours.

By contrast to observations in offspring from HFD-fed dams, HFD treatment applied during early life had a minor impact on the VTA-NAc DA pathway. For example, HFD treatment produced no significant changes in DA neuronal function of the VTA, DA release in the NAc, and it generated contrasting variations in the expression of DAT and DA receptors [108,113]. Further work is therefore needed to characterise the impact of HFD treament impact the activity of the mesolimbic DA system.

#### 5.4.2. Combined Effecs of a High-Fat Diet Treatment with Psychostimulant Drugs

When combined with psychostimulant drugs, HFD treatment significantly increased the activity of VTA DA neurons in response to amphetamine; it also potentiated NAc DA release and induced the recruitment of postsynaptic DA receptors, which resulted in increased amphetamine-induced locomotor activity in HFD-fed animals [113]. In the NAc and the striatum, a juvenile HFD treatment modulated the expression of the cannabinoid receptor 1 and the opioid mu receptor 1 [107,110], which may reflect variations in the release of endogenous peptides. Together, the results provided in these studies suggest that the neuroadaptations in the brain reward pathway in HFD-fed animals may contribute to the changes observed in psychostimulant-related behaviour.

### 5.5. Conclusions

Taken together, the reviewed data from the literature call for further studies aiming at understanding the relationship between the effects of HFD treatment in different developmental periods on behavioural endpoints and the changes in neurotransmission and molecular plasticity in specific brain areas. In addition, further work is crucially needed to characterise the impact of HFD treatment on VTA–NAc signalling elements that play pivotal roles in reward/reinforcement circuits of the mesolimbic system. Because the homeostatic and reward circuits are often studied separately, the impact of HFD treatment on reward circuits is a question that remains mostly unanswered.

## 6. Combined Impact of High-Fat Diet and Stress Exposure in Rodents

**Figure 1:**
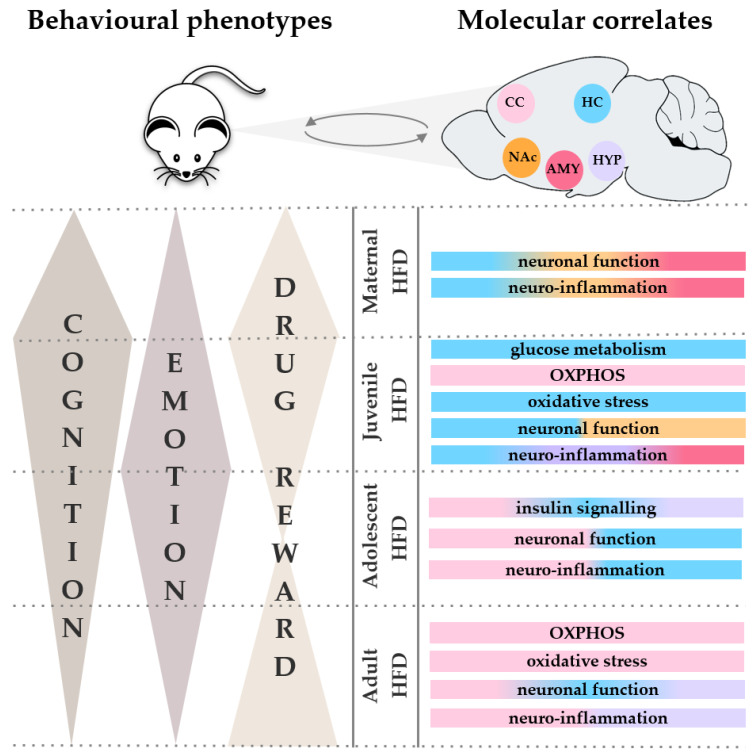
Overview of the impact of a high-fat diet (HFD) treatment on behavioural phenotypes (i.e., emotionality, cognition and reward-related behaviour) in rodents, as well as associated brain mechanisms. Treatment with a HFD leads to alterations in energy metabolism (i.e., glucose metabolism, insulin signalling), molecular correlates of mitochondrial functions (e.g., OXPHOS, oxidative stress and neuroinflammation) and neuronal function (i.e., neurotransmission, synaptic plasticity) in a subset of brain areas, depending on the developmental period at which the HFD is administered. Abbreviations: AMY: amygdala; CC: cerebral cortex; HC: hippocampus; HFD: high-fat diet; HYP: hypothalamus; NAc: nucleus accumbens.

As reviewed above, HFD treatment (25–50% kcal from fat, predominantly 45%) in rodents produced some impairments in emotionality, cognition and reward-related behaviour, which were accompanied by distinctive neuroanatomical and molecular changes according to the period in which the HFD treatment was applied (see summary in Figure 1). However, these observations were made under basal, non-stressed conditions.

Because both poor dietary choices and stress can lead to disruptions in emotional behaviour, cognitive performance and reward-related behaviour, it is important to consider their effects combined.

The analysis of the literature examining the impact of HFD treatment in animals exposed to chronic stress and showing depression-related phenotypes reveals mixed findings. Some studies reported that adolescent stress and ad libitum HFD interacted to produce a greater vulnerability to the detrimental effects of HFD on emotionality and reward sensitivity in male rodents exposed to psychological stressors. For example, exposure to predator odour threat increased anxiety-related behaviours in HFD-fed rats and reduced brain volume, especially the hippocampal volume, compared to HFD-fed unstressed rats [152]. In another study, HFD-fed mice subjected to a vicarious social defeat stress, wherein one mouse witnesses the physical defeat of a conspecific from the safety of an adjacent compartment (i.e., uncoupling of emotional and physical stress), showed increased social avoidance and reduced hedonic response [153].

Some studies reported that the interaction of stress and HFD reduced the vulnerability to the unfavourable effects of HFD on behaviour and energy metabolism in male rodents exposed to combined psychological and physical stressors. For example, HFD-fed adult mice exposed to social defeat stress showed a reduced body weight gain compared to HFD-fed unstressed mice despite a significant increased caloric intake, an effect due to increased energy expenditure and especially increased fat oxidation [154]. As another example, limited access to HFD treatment reduced the anxiety-related behaviour of adolescent rats subjected to social defeat stress as well as changed their behaviour during the direct confrontation with residents [155]. Together, these two studies confirm the stress-buffering/comfort properties of palatable food against stressors [156].

It is important to mention that a few studies fail to observe an interaction of stress and diet in rodents. For instance, HFD-fed male mice exposed to early life stress in the form of neonatal maternal separation or to unpredictable chronic mild stress showed, in adulthood, no significant differences in metabolic parameters [157] or self-care behaviour and hedonic response [158] compared to HFD-fed unstressed mice.

Taken together, these studies highlight that the chronic activation of the stress response in rodents interfered with HFD treatment to produce behavioural and metabolic impairments that depended on the type and the duration of stress exposure. Interestingly, HFD treatment could reduce the antidepressant efficacy of fluoxetine, a selective serotonin reuptake inhibitor, on stress-induced depression-like behaviour [153,158]. These data suggest that diet plays a role in antidepressant efficacy and parallel some clinical findings showing poor responsiveness to antidepressant therapy in patients suffering from obesity and related metabolic disorders [159,160].

## 7. Discussion

### 7.1. Overview of the Impact of a High-Fat Diet Treatment on Behaviour and Brain in Rodents

In the present review, we have provided an overview of important behavioural and molecular correlates of HFD-induced impairments in rodents (see Figure 1). While the use of varying diets, developmental stages, and behavioural tests make the comparison between studies difficult, HFD treatment generally produced a long-lasting detrimental impact on emotionality, cognition and psychostimulant-related behaviours (see summary in Table 1). In addition, HFD treatment produced marked alterations in neuroplasticity and neuro-inflammation, but only slight impairments in energy metabolism and mitochondrial functions (see summary in Table 2). However, only a few studies have measured the effects of HFD treatment on brain energy metabolism and plasticity in the attempt to link behavioural alterations with molecular phenotypes. In addition, when molecular studies were performed, they were limited to one brain area or a small number of brain regions. Therefore, the molecular, cellular, and neural mechanisms underlying the HFD-induced alterations in emotionality, cognition and reward-related behaviour remain to be elucidated.

### 7.2. Limitations

#### 7.2.1. Lack of Systematic Assessment of the Metabolic Status

One important point to consider is that the assessment of dietary-induced alterations in the peripheral metabolic status was not systematic (see Supplementary Table S1). Therefore, it is unclear whether the HFD-induced changes in brain and behaviour result from the dietary manipulation (i.e., the diet, the diet composition or the high caloric density) or are instead the consequence of metabolic dysfunctions that develop with weight gain, adiposity and peripheral/central metabolic changes. In fact, a few studies observed that HFD treatment induced alterations in behaviour without producing adverse metabolic effects. For example, both ad libitum and restricted HFD treatments produced major changes in psychostimulant-related behaviours without increasing the body weight and altering the insulin and leptin signalling [107,108,110,161].

While the verification of the body weight gain has performed almost consistently, few studies have investigated whether HFD treatment produced insulin resistance, as measured by elevated fasting plasma glucose and insulin, and the measure of leptin levels has also not been routinely done (see Supplementary Table S1). Because insulin and leptin have a critical role not only in whole-body energy homeostasis but also in regulating neuronal structure and function (e.g., through regulation of synaptic plasticity and trafficking of neurotransmitter receptors) [162,163], further work is needed to link behavioural alterations with changes in insulin and leptin signalling in brain areas associated with emotionality, cognition and reward. Because of the close interaction of mitochondria, leptin and insulin signalling in the brain, a better understanding of the underlying mechanisms by which insulin and leptin resistance modify mitochondrial functions may help identify novel therapeutic strategies to combat obesity and associated comorbidities.

#### 7.2.2. Lack of Evaluation of the Neuroendocrine and Immune Systems

It is also important to highlight that relatively little is known about the impact of HFD treatment on neuroendocrine function and peripheral mediators of inflammation from the studies reviewed. In fact, contrasting effects of HFD treatment were found on corticosterone release in offspring of HFD-fed dams [89,90,145,164] and HFD treatment had no major impact on corticosterone release in HFD-fed animals [102,107,112,120,127]. In addition, discrepant findings were found on peripheral measures of oxidative stress and inflammation [101,114,144,147]. Further investigation is needed to determine whether and by which mechanisms HFD treatment could disturb hypothalamic–pituitary–adrenal axis function and produce systemic immune dysregulation.

Although the description of the effects of HFD treatment on gut microbiota falls out of the scope of this review, it appears important to highlight the importance of this topic. It is well documented that the food consumed affects the bacteria composition within the gut microbiome, which plays a crucial role in food absorption, nutrient and energy extraction as well as low-grade inflammation [56]. In particular, high-calorie diets and specific dietary components play a role in shifting the microbiota composition (e.g., alteration of gut integrity, community profiling or metabolite production, inflammation) [56]. Such changes in gut microbiome composition have been recently shown to contribute, in rodents, to diet-induced anxiety- and depression-like behaviour as well as cognitive impairments, especially in hippocampus-dependent tasks [57]. Thus, understanding the roles of the gut microbiota in emotionality, cognition and reward may hold exciting prospects for the treatment of obesity and associated comorbidities (e.g., SUD).

#### 7.2.3. Lack of Studies on the Sensitivity to Drugs of Abuse and Dopaminergic Neurotransmission

Surprisingly, the impact of HFD on psychostimulant-related behaviours has been very scarcely studied, despite the substantial evidence that obesity and SUD share common biological substrates [18]. Only a few rodent studies have investigated the HFD-induced alterations in addiction-relevant behaviours and the associated cellular and molecular mechanisms in reward-related areas (see Table 1 and Supplementary Table S1). Consequently, much remains to be investigated with respect to the behavioural evidence and underlying mechanisms of the interaction between HFD and psychostimulant drugs. Importantly, both the schedule of HFD consumption and the social housing conditions (single- versus group-housed animals) seem to modulate the outcomes of psychostimulant-related behaviours. A recent study suggested that the post-weaning housing condition could modulate the susceptibility to HFD-induced body weight gain, adiposity and thermoregulation in male mice [165], highlighting the need to consider the housing density in the interpretation of the behavioural and metabolic data.

Interestingly, some neuroadaptations produced by HFD treatment parallel those observed with cocaine (see summary in Supplementary Table S2). For example, cocaine exposure produced slight changes in glucose and phospholipid metabolism as well as neurotransmission and increased oxidative stress in the NAc [166,167]. In addition, similar to observations made with HFD treatment [144], cocaine exposure increased oxidative stress and reduced mitochondrial activity in isolated synaptosomes from the whole brain [168]. Moreover, it produced significant changes in the gene expression pattern of electron transport chain complexes in the cingulate cortex, which connects the prefrontal cortex and the limbic system [169,170]. Because the mitochondrial DNA (mtDNA) copy number was reported to be increased in the prefrontal cortex and hippocampus of rats with a history of cocaine self-administration [170], perhaps the variation in mtDNA compensated a decreased number of mitochondria or mitochondrial synthesis in order to maintain a normal level of mitochondrial transcription to provide ATP [171]. A history of cocaine self-administration induced neuroplasticity in the NAc through alterations in the pattern of genes important in several mitochondrial functions (e.g., mitochondrial transcription and replication, mitochondrial dynamics and energy production) [166,172,173]. The possible mechanisms underlying the changes in molecular correlates of mitochondrial dysfunction by cocaine have yet to be determined, but they may occur in response to the massive DA release due to cocaine binding to transporter sites of monoamines, as well as from the oxidative potential of cocaine metabolites [49].

Importantly, cocaine oppositely modulated the accumbal mitochondrial morphology and the mRNA expression of peroxisome proliferator-activated receptor gamma coactivator 1-alpha (Pgc1α), a master regulator of mitochondrial biogenesis, and dynamin-1 like (Drp1), a regulator of mitochondrial division, in dopamine receptor D1-expressing medium spiny neurons (D1-MSNs) and dopamine receptor D2-expressing MSNs (D2-MSNs) [166,172,173]. Because cocaine has been reported to produce contrasting effects on dendritic spine density, excitatory plasticity, signalling processes and transcriptional activity in D1-MSNs and D2-MSNs [174,175], further work is needed to understand the importance of mitochondria in cocaine behavioural plasticity in specific neuronal subtypes.

#### 7.2.4. Lack of Studies on Sex Differences

Apart from the studies investigating the effects of a maternal HFD treatment in dams and/or their offspring, male rodents have typically been used to examine the behavioural and molecular effects of HFD treatment. Thus, only very few studies have examined the effects of HFD treatment in female rodents. Therefore, sex differences were not explicitly addressed in this review. One study found an increased anxiety-related behaviour in HFD-fed female mice [176], but the experimental design lacked a direct comparison between the sexes. Another study found no signs of molecular alterations in the hypothalamus of HFD-fed female and male rats [148]. Interestingly, Rodenas-Gonzalez and colleagues observed that certain patterns of HFD treatment blocked the reinstatement of cocaine-induced CPP in male mice but not in female mice, suggesting that female animals might be less sensitive to the protective effects of HFD treatment [110]. Given the global gender disparities in stress-related disorders [177] as well as obesity and metabolic diseases [178,179,180], more studies in female rodents are imperative to determine the possible sex differences in response to dietary intervention.

#### 7.2.5. Remaining Challenges in Translating Rodent High-Fat Diet Treatments to Human Obesity

Finally, it is worth mentioning that we have reviewed preclinical studies that used a forced exposure of rodents to HFD treatment. While such dietary manipulation allows studying the behavioural, physiological and neural responses to unhealthy diets, this deviates from the human situation where individuals can make food choice. In addition, it does not mimic the importance of the high caloric intake in the form of fluids in humans. Studies that examined how HFD composition, pattern and form (i.e., solid/liquid) affects behaviour and associated brain function and plasticity are lacking. Available studies show that rodents given free choice to consume a high-energy diet typically show hyperphagia, snacking behaviour and increased food-motivated behaviour, leading to accelerated behavioural and metabolic alterations similar to those observed in human obesity [181,182,183,184,185,186,187]. Interestingly, strong sex differences in food choice were observed in adult mice that were exposed to an early life stress paradigm (i.e., limited nesting and bedding material) combined with acute stress exposure in adulthood [188]. The preference for fat in female mice was accompanied by sex differences in the physiological stress response as well the brain circuits regulating food intake and reward [188]. Given the importance of the issue of sex differences in stress responses, further research is warranted and clearly needed to determine the sex-specific motivational aspects of HFD feeding and the underlying precise mechanistic bases.

## 8. Conclusions

In conclusion, our in-depth analysis of the published literature revealed that high-energy diets had a pronounced impact on emotionality, cognition and reward-related behaviour as well as underlying brain processes that depended on the developmental period. Because most of the studies reviewed involved only male rodents (apart from those examining the effects of a maternal HFD treatment in dams and/or their offspring), future research should extend these observations to female rodents. This would help to elucidate the sex-specific mechanisms linking stress and obesity. Profound knowledge about the molecular mechanisms linking stress and obesity is crucially needed in translational research using rodent models and can significantly advance the important search for risk-biomarkers and the development of clinical intervention strategies.

## Figures and Tables

**Table 1:** Literature overview of the impact of a high-fat diet treatment on emotionality, cognition and reward-related behaviours in rodents, taking into account the developmental period. Description: Studies are organised by the developmental period (i.e., before puberty or early adolescence, during late adolescence or in adulthood) in which the high-fat diet (HFD) treatment was applied. For the effects of a maternal HFD treatment, studies are organised by the developmental period (i.e., before puberty or early adolescence, during late adolescence or in adulthood) in which the offspring were tested. All studies reviewed used male (♂) mice (m) or rats (r), otherwise indicated by the female (♀) symbol. The numbered references refer to the bibliography section.

Behavioural Test	Assessement	Outcomes & Ref.			
		⬆ (Increased)	⬇ (Decreased)	⬌ (Not Changed)	Conclusion
**Maternal HFD treatment (pre-partum)** **Testing in adult offspring**					
EPM	Anxiety			♂♀ (pooled) [83r]	⬌ (not changed)
OFT	Locomotion/exploration			♂♀ (pooled) [83r]	⬌ (not changed)
OBT	Learning & memory		♂♀ (pooled) [83r]		⬇ (decreased)
**Maternal HFD treatment (pre- and post-partum)** **Testing in juvenile offspring**					
EZM	Anxiety			♂♀ [86r]	⬌ (not changed)
FST	Passive stress-coping	♂♀ [86r]			⬆ (increased)
AC	Locomotion/exploration			♂♀ [86r, 87r]	⬌ (not changed)
RW	Volontary exercise	♀ [85r]	♂ [85r]		Δ (sign. changes)
SPT	Anhedonia			♂♀ [86r]	⬌ (not changed)
NORT	Learning & memory			♂♀ [86r]	⬌ (not changed)
AC	AMPH locomotion		[87r]		⬇ (decreased)
AC	AMPH sensitization		[87r]		
**Maternal HFD treatment (pre- and post-partum)** **Testing in adolescent offspring**					
AC	Locomotion/exploration			♀ [88r]	⬌ (not changed)
ACQ	COC- self-administration			♀ [88r]	⬌ (not changed)
EXT				♀ [88r]	
RST				♀ [88r]	
**Maternal HFD treatment (pre- and post-partum)** **Testing in adult offspring**					
EZM	Anxiety			♂♀ (pooled) [83r]	⬌ (not changed)
AC/OFT	Locomotion/exploration			♂♀ (pooled) [83r, 145r]	⬌ (not changed)
OBT	Learning & memory			♂♀ (pooled) [83r]	⛒ (inconcl.)
MWM				[89r]	
**Juvenile HFD treatment**					
EPM/ETM/MBT/ OFT/DaLi	Anxiety	[92r, 100r, 101m, 103r, 104m]	[104m]	[93m, 94m, 95m, 99m, 102m, 104m]	⛒ (inconcl.)
FST	Passive stress-coping	[92r]	[95m, 105r]	[101m, 102m, 106r]	⛒ (inconcl.)
OFT/CA	Locomotion/exploration			[93m, 94m, 95m, 100r, 101m, 102m,103r]	⬌ (not changed)
HBT			[101m]		
FUST/SIT	Anhedonia			[102m]	⬌ (not changed)
NORT/RAM/HWM/	Learning & memory		[93m, 94m, 95m, 96m]		⬇ (decreased)
MWM/PAT				[92r, 109m]	
EXP	COC/AMPH-ind. CPP	[112m *$]	[108r *, 109m, 112m *$]	♂♀ [107m *, 108r, 109m, 110m *]	Δ (sign. changes)
EXT				♂♀ [107m *, 110m *, 112m *]	
RST		[99m]	[110m *]	[107m *, 112m *]	
ACQ/RST	COC self-administration	[107m *]			⬆ (increased)
OFT/AC	COC/AMPH locomotion			[99m, 113r]	Δ (sign. changes)
AC	AMPH sensitization	[113 r]	[108r]		
EPM	COC-ind. anxiety	[107m *]			
FST	COC-ind. immobility		[106r]		
**HFD treatment in late adolescence**					
EPM/EZM/MBT/OFT/ NSF/DaLi	Anxiety	[114r, 115m, 118m, 177m]		[116m, 120m]	⬆ (increased)
FST/TST	Passive stress-coping	[117m]		[102m, 116m, 118m]	⛒ (inconcl.)
OFT/TM	Locomotion/exploration		[117m, 119m]		⬇ (decreased)
SPT/SxB/FUST	Anhedonia	[116m]		[102m]	⛒ (inconcl.)
SIT		[116m]		[120m]	
SRT			[116m]		
NORT/MWM/BM	Learning & memory			[116m, 121m, 122m]	⬌ (not changed)
OBT			[122m]		
**Adult HFD treatment**					
OFT	Anxiety			[126m]	⬌ (not changed)
OFT/YM	Locomotion/exploration		[127m]	[126m]	⛒ (inconcl.)
SPT	Anhedonia	[125r]			⬌ (not changed)
TWAT	Learning & memory			[126m]	⬌ (not changed)
OFT/AC	COC/AMPH locomotion	[129r]	[128r]		Δ (sign. changes)
EPM	COC-ind. anxiolysis			[128r]	
FST	COC-ind. active coping	[128r]			

Abbreviations: AC: activity cage; ACQ: acquisition; AMPH: amphetamine; BM: Barnes maze test; COC: cocaine; CPP: conditioned place preference test; DaLi: dark–light box test; EPM: elevated plus-maze test; ETM: elevated T-maze test; EZM: elevated zero-maze test; EXP: expression; EXT: extinction; FST: forced swim test; FUST: female urine sniffing test; HBT: hole board test; HFD: high-fat diet; HWM: Hebb Williams maze test; m: mice; ind.: induced; MBT: marble burying test; MWM: Morris water maze test; NORT: novel object recognition test; NSF: novelty-suppressed feeding test; OBT: operant bar-pressing task; OFT: open-field test; PAT: passive avoidance task; r: rats; RAM: radial arm maze test; RST: reinstatement; RW: running wheel; SIT: social interaction test; SPT: sucrose preference test; SRT: social recognition test; SxB: sexual behaviour; TM: T-maze test; TST: tail suspension test; TWAT: two-way avoidance task; YM: Y-maze test. Symbols: * limited access to a high-fat diet; $: outcomes differing based on the housing conditions.

## Data Availability

Not applicable.

## Supplementary Materials

The following supporting information can be downloaded at: https://www.mdpi.com/article/10.3390/ijms23147952/s1.
