# Supplementary material for: Neurobiological Mechanisms Modulating Emotionality, Cognition and Reward-Related Behaviour in High-Fat Diet-Fed Rodents
Source: Int J Mol Sci. 2022 Jul 19;23(14):7952. doi: 10.3390/ijms23147952 (PMC9317076; doi:10.3390/ijms23147952)
Supplement: Supplementary file 1 [file ijms-23-07952-s001.zip › ijms-1760851-supplementary.pdf]

Supplementary Information

Neurobiological mechanisms modulating emotionality, cognition and reward-related behaviour in high-fat diet-fed rodents

Dorothea Ziemens[1,2], Chadi Touma[1], Virginie Rappeneau[1*i]

[1]Department of Behavioural Biology, University of Osnabrueck, Barbarastrasse 11, 49076 Osnabrück, Germany

[2]Institute for Experimental and Clinical Pharmacology and Toxicology, Center of Brain, Behavior and Metabolism, University of Luebeck, Ratzeburger Allee 160, 23562 Luebeck, Germany

*Correspondence: Email: virginie.rappeneau@uni-osnabrueck.de; Fax: +49 (0)541 969 2862

Running title: High fat diet, psychostimulants and brain energy metabolism

Keywords: rodent, high-fat diet, obesity, psychostimulant drugs, depression, anxiety, energy metabolism, mitochondria, oxidative stress, neuroplasticity

Supplementary Table S1. Effects of a high-fat diet treatment on behaviour and brain.

Studies are organised by the developmental period at which the high-fat diet (HFD) was applied in rodents (i.e., before puberty and during early adolescence, during late adolescence or in adulthood) as well as by duration of the dietary challenge. For studies on the effects of a maternal HFD treatment, studies are organised by the developmental period (i.e. before puberty or early adolescence, during late adolescence or in adulthood) at which the offspring were tested. The column "Design" refers to the rodent sex, strain, species and age at which the HFD was applied. It also indicates the duration of the HFD (vs. control diet) in weeks as well as the fat content of the HFD and control diets. When information was available, the precise reference of the diet and its supplier are mentioned. Nomenclature for genes (lower cases, italicised) and proteins (upper cases) is respected in the table. The numbered references refer to the bibliography section placed at the end of the supplementary material.

Ref	Design	Metabolism	Test	Findings	Tissue sampled	Findings
	**HFD treatment during pregnancy – effects in dams**					
[1]	Juvenile dam ♀ Wi<br>From PND21-90<br>For 3 W:<br>HFD 25% fat<br>(ref. & supplier ns)<br>CON 5% fat<br>(RQ 22-5, Zeigler Rodent)	↑ CORT	-	-	-	-
[2]	Juvenile dam ♀ SD<br>From 8 W<br>For 3 W:<br>HFD 32.5% fat<br>(D12266B, Open Source Diets)<br>CON 12.3% fat<br>(3436, Provimi Kliba)	↑ BW<br>↔ insulin<br>↑ leptin<br>↑ glucose	-	-	-	-
	Juvenile dam ♀ SD<br>From 8 W (Gen. F3)<br>For 3 W:<br>HFD 32.5% fat<br>(D12266B, Open Source Diets)<br>CON 12.3% fat<br>(3436, Provimi Kliba)	[↔] BW in DIO/DR<br>↔ insulin in DIO/DR	-	-	-	-
	**HFD treatment during pregnancy – effects in adult offspring**					
[1]	♂♀ (pooled)<br>Offspring Wi<br>adult PND75-80<br>fed CON 5% fat from P21<br>Derived from ♀ Wi<br>From PND21-90<br>For 3W:<br>HFD 25% fat<br>(ref. & supplier ns)<br>CON 5% fat<br>(RQ 22-5, Zeigler Rodent)	-	OFT<br>EPM<br><br>OBT	[↔] total distance<br>↔ time, entries in open arms<br>↑ number of sessions (FR)<br>↔ number of rewards (PR)	-	-
	**HFD treatment during pregnancy and lactation – effects in dams**					
[3]	Juvenile dam ♀ B6<br>From 3W<br>For 6 W:<br>HFD 45% fat<br>(D12451, RD)	↔ BW<br>↔ glucose tolerance	MB	↔ nest pup retrieval<br>Δ nest building, exploration behaviour, in nest with pups/moving	-	-

	CON 10% fat<br>(D12450H, RD)					
[1]	Juvenile dam ♀ Wi<br>From PND21-90<br>For 6 W:<br>HFD 25% fat<br>(ref. & supplier ns)<br>CON 5% fat<br>(RQ 22-5, Zeigler Rodent)	↑ CORT	-	-	-	-
[2]	Juvenile dam ♀ SD<br>From 8W<br>For 6 W:<br>HFD 32.5% fat<br>(D12266B, Open Source Diets)<br>CON 12.3% fat<br>(3436, Provimi Kliba)	↑ BW<br>↔ insulin<br>↑ leptin<br>↑ glucose	MB	↔ latency, duration of nest pup retrieval<br>↔ latency, duration of nursing behaviour	-	-
	Juvenile dam ♀ SD<br>From 8 W (Gen. F3)<br>For 6 W:<br>HFD 32.5% fat<br>(D12266B, Open Source Diets)<br>CON 12.3% fat<br>(3436, Provimi Kliba)	↑ BW in DIO/DR<br>↔ insulin in DIO/DR	MB<br><br><br><br><br>OFT<br>SPT	↔ latency, duration of nest pup retrieval<br>↑ latency of nursing behaviour<br>↔ duration of nursing behaviour<br>↔ total distance<br>↔ sucrose preference	-	-
[4]	Adult dam ♀ Wi<br>From P110<br>For 6 W:<br>HFD 45% fat<br>(D12451, RD)<br>CON 10% fat<br>(D12450K, RD)	↔ BW<br>↑ adiposity	MB	↓ licking, grooming<br>↔ contact with pups	-	-
			**HFD treatment during pregnancy and lactation – effects in offspring**			
[3]	♂♀ Offspring – pups<br>PND3<br>Derived from ♀ B6<br>From 3W<br>For 6W:<br>HFD 45% fat<br>(D12451, RD)<br>CON 10% fat<br>(D12450H, RD)	↔ BW<br>↑ glucose tolerance	-	-	-	-
[5]	♂ Offspring –pups<br>PND1-10<br>Derived from ♀ SD<br>age ns (from last week G)<br>For 4W:<br>HFD 30% fat<br>CON 5% fat<br>(ref. ns, Harlan)	↔ BW (P1)<br>↑ BW (P10)<br>↑ adiposity<br>↑ leptin<br>↔ insulin<br>↑ CORT (basal, stress)<br>↔ ACTH (basal, stress)	-	-	-	-
[4]	♂♀ Offspring – pups<br>PND1-21; adult P105<br>CON 10% fat from P21<br>Derived from ♀ Wi<br>From 11W<br>For 6W:<br>HFD 45% fat<br>(D12451, RD)<br>CON 10% fat	↓ BW (P1-P9)<br>↑ BW (P21)<br>↑ adiposity<br>↔ glucose, leptin (P1)<br>↓♂,↔♀ insulin (P1)<br>↔♀, ↓ ♂ glucose (P21)<br>↑ leptin (P21)	-	-	-	-

	(D12450K, RD)	↓♀,↔ ♂ insulin (P21)<br>Δ reproductive function (P105)				
[6]	♂♀ Offspring – juvenile 60 days<br>*Cross-fostering*<br>CON 12% fat from P21<br>Derived from ♀ SD<br>From 11-12W<br>For 6W:<br>HFD 25% fat<br>(ref. & supplier ns)	↓ BW (P1)<br>↔ BW (W1-W7)<br>↔ glucose<br>↔ insulin<br>Δ plasma lipids	RW	↓ ♂, ↑ ♀ number of wheel turns	STR	↔ D2R
[7]	♂ Offspring – juvenile PND25, 45<br>Adult PND95<br>CON 5% fat from P21<br>Derived from ♀ SD<br>From 6-7W<br>For 6W:<br>HFD21% fat CON 5% fat<br>(ABDiet)	↔ BW (P1)<br>↑ BW (P21- P89)<br>↑ adiposity<br>↔ glucose, insulin, leptin	FPT	↑ preference for 1% corn oil solution (P25), ↔ P95	NAc	↔ TH density fibres (P25,P45)<br>↓ TH density fibres and mRNA *Th* (P95)<br>Δ gene expression (plasticity, ↑ 5HT1R) (P95)<br>↑ mRNA *D2r* (P25)
					VTA	↓ TH positive neurons and mRNA *Th* (P45)<br>↔ TH positive neurons and mRNA *Th* (P25,P95)<br>Δ gene expression (plasticity, ↑ 5HT1R) (P95)
					HYP	Δ gene expression (plasticity, ↑ 5HT1R) (P95)
[8]	♂♀ Offspring – juvenile PND28-34<br>CON 13% fat from P21<br>Derived from ♀ Wi<br>From 8W<br>For 6W:<br>HFD 28.5% fat<br>(C1011, Altromin)<br>CON 13 % fat<br>(VRF1, Special Diets Service)	-	AC<br>EZM<br><br>FST<br><br>SPT<br>NORT	↔ total distance<br>↔ time, entries in open arms<br>↑ immobility, ↓ swimming<br>↔ sucrose preference<br>↔ recognition index	FCx	Δ gene expression (excitatory and inhibitory cortical neurons)<br>↑ expression of genes involved in intracellular transport-related processes (cytoskeleton-dependent intracellular transport, synaptic vesicular transport and endocytosis)<br>↓ expression of genes involved in basic cellular function aspects (cellular respiration and protein, translation and degradation)
[9]	♂ Offspring – juvenile PND56-62<br>CON 5% fat from P21<br>Derived from ♀ SD<br>age ns (from last week G)<br>For 4W:<br>HFD 30% fat<br>CON 5% fat<br>(ref. & supplier ns)	↔ BW (P1)<br>↑ BW (P11)<br>↔ BW (P25, P90)	AC	↔ beam interruptions (basal locomotion)<br>↓ AMPH-induced locomotion (0.75 mg/kg i.p.)<br>↓ AMPH-induced sensitization (3*0.75 + 5*1.5 + challenge 0.75 mg/kg i.p.)	NAc<br>STR<br>PFC<br>VTA	↑ DA, DOPAC; ↔ D1R, D2R, DAT, TH<br>↔ D1R, D2R, DAT, TH<br>↔ DA, DOPAC<br>↑ TH
[10]	♀ Offspring – adolescent PND63<br>CON 13% fat from P21<br>Derived from ♀ Wi<br>From 8W<br>For 6W:<br>HFD 28.5% fat<br>(C1011, Altromin)<br>CON 13 % fat<br>(VRF1, Special Diets Service)	↔ BW	AC<br>SA	↔ total distance<br>↔ cocaine-induced SA (0.25-1 mg/kg i.v.; FR, PR)<br>↔ cocaine-induced EXT<br>↔ cocaine-induced RST	NAc<br>PFC/ STR/ HYP/ VTA	↑ MC-4R (synaptosomes)<br>↔ MC-4R (synaptosomes)

[8]	♂♀ Offspring – adolescent PND63-69<br>CON 13% fat from P21<br>Derived from ♀ Wi<br>From 8W<br>For 6W:<br>HFD 28.5% fat<br>(C1011, Altromin)<br>CON 13 % fat<br>(VRF1, Special Diets Service)	-	AC<br>EZM<br><br>FST<br><br>SPT<br>NORT	↔ total distance<br>↔ time, entries in open arms<br>↔ immobility, ↓ swimming<br>↔ sucrose preference<br>↔ recognition index	-	-
[1]	♂♀ Offspring – adult PND75<br>CON 5% fat from P21<br>Derived from ♀ Wi<br>PND21:90<br>For 6W:<br>HFD 25% fat<br>(ref. & supplier ns)<br>CON 5% fat<br>(RQ 22-5, Zeigler Rodent)	-	OFT<br>EPM<br><br>OBT	↔ total distance<br>↔ time, entries in open arms<br>↔ number of sessions (FR)<br>↔ number of rewards (PR)	-	-
[11]	♂♀ Offspring adult 12-20 W<br>CON 10% fat from P21<br>Derived from ♀ Wi<br>From PND110<br>For 6W:<br>HFD 45% fat<br>CON ns<br>(ref. ns, Harlan)	↔ BW<br>↔ glucose<br>↑ leptin<br>↔ CORT	OFT	↔ total distance travelled, time in centre	HC (CA1)<br>AMY	↓ dendritic length, ↔ spine density<br>↓ dendritic length, ↔ spine density
[12]	♂ Offspring – adult 15-16W<br>CON 5.3% fat from P21<br>Derived from ♀ SD<br>From PND30-35<br>For 6W:<br>HFD 25% fat<br>CON 5.3% fat<br>(Mediscience Ltd)	↑ CORT (basal, acute restraint stress, LPS)<br>↔ CORT (insulin)<br>↔ CORT (chronic restraint stress)	-	-	HYP (PVN)<br>AMY	↑ mRNA *Crf* (basal, chronic stress), ↔ mRNA *Crf* (acute stress)<br>↑ mRNA *Nr3c1*, *Nr3c2*
[13]	♂ Offspring –adult 20W<br>HFD 39% or CON 12% fat from P21-20W<br>Derived from ♀ SD<br>From age 6:7W<br>For 6W:<br>HFD 39% fat<br>CON 12% fat<br>(ref. ns, ABDiet)	↔ BW (P1)<br>↑ BW (P21)<br>↔ glucose, insulin<br>Δ plasma lipids<br>↔ CORT	MWM	↓ % platform crossing<br>↔ latency to reach the platform (HFD/HFD)<br>↔ % platform crossing, latency to reach the platform (HFD/CON)	HC	↓ DCX-positive cells (HFD/HFD)<br>↔ DCX-positive cells (HFD/CON)<br>↔ astrocytic process number and total length (HFD/HFD)<br>↑ astrocytic process number and total length (HFD/CON)
**HFD before puberty and during early adolescence**						
[14]	♂♀Wi<br>From 3W<br>For 5-6W:<br>HFD 42% fat<br>CON 4% fat<br>(ref. ns, Nuvilab®)	↔ glucose<br>Δ lipids	-	-	HYP	↔ ETC CI-V<br>↔ MT mass<br>↔ MMP<br>↔ SOD, CAT, DCF<br>↔ MDA
[15]	♂B6<br>From 4W	↑ BW	DaLi<br>MBT	↔ time in light zone<br>↑ marbles buried	HC	↑ mRNA *IL10*<br>↔ mRNA *IL1β*, *IL6*, *TNFα*

	For 8W:<br>HFD 45% fat<br>(D12451, RD)<br>CON 10% fat<br>(D12450B, RD)				 AMY<br>HYP	↔ IBA1-immunoreactivity<br>↑ IBA1-immunoreactivity<br>↔ IBA1-immunoreactivity
[16]	♂B6<br>From 4W<br>For 8W:<br>HFD 45% fat<br>(D12451, RD)<br>CON 10% fat<br>(D12450H, RD)	↑ BW<br>↑ adiposity<br>↑ leptin<br>↔ ADPN<br>Δ lipids	-	-	Blood<br>HYP	↔ IFNγ, IL5, IL10, IL6, TNFα, IL1 β<br>↑ mRNA *Insr*, ↔ mRNA *Irs1, Mtor, Tgfβ*<br>↓ mRNA *Nd*, ↔ mRNA *Cytb*<br>↔ ATP content<br>↔ MT ratio types, aspect ratio, surface coverage, ↓ MT area<br>↔ TAS<br>↑ ARC IBA1-immunoreactivity<br>↔ PVN IBA1-immunoreactivity
[17]	♂B6<br>From 5W<br>For 8W:<br>HFD 45% fat<br>(D12451, TestDiet)<br>CON 18% fat<br>(2018, Harlan Teklad)	-	EPM<br>FST<br>OFT<br><br>NORT	↔ time in open arms<br>↓ time immobile<br>↔ squares crossed, rearing<br>↓ discrimination index	-	-
[18]	♂CD1<br>From 5W<br>For 8W:<br>HFD 45% fat CON 10% fat<br>(ref. ns, RD)	↑ BW<br>↑ leptin<br>↔ insulin<br>↔ glucose<br>Δ lipids	HBT<br>FST<br>OFT	↓ head dips<br>↔ time immobile<br>↔ total distance<br>↓ time in centre	blood	↔ GSH, GSSG, GSH/GSSG
[19]	♂ B6<br>From 5W<br>For 8W:<br>HFD 45% fat<br>(D12451, RD)<br>CON 10% fat<br>(D12450B, RD)	↑ BW<br>↑ adiposity<br>↑ leptin<br>↔ insulin<br>↔ glucose<br>↔ lipids	RAM	↑ time to find baited arm, ↑ number of errors	-	-
[20]	♂WH<br>From 3W<br>For 10W:<br>HFD 45% fat<br>(D12451, RD)<br>CON 3% fat<br>(RF21, Mucedola)	↑ BW<br>glucose intolerance<br>↑ CORT	EPM<br>FST<br>MWM	↓ time in open arms<br>↑ time immobile<br>↔ distance swum to reach platform	-	-
[21]	♂B6<br>From 3W<br>For 15W:<br>HFD 45% fat<br>CON 10% fat<br>(ref. ns, RD)	↑ BW<br>↑ adiposity<br>↑ leptin<br>↑ insulin<br>↔ CORT	DaLi<br>FST<br>FUST<br>OFT<br>SIT	↔ time in light zone<br>↔ time immobile<br>↔ time sniffing urine<br>↔ total distance<br>↔ time exploring mouse	-	-
[22]	♂OF1<br>From 4W<br>For 10W:<br>HFD 45% fat<br>(TD.06415, Harlan Teklad)<br>CON 13% fat<br>(2014, Harlan Teklad)	↑ BW<br>↑ leptin	EPM<br>OFT<br>PAT<br><br>HWM	↔ time in open arms<br>↔ total distance<br>↑ latency to aversive zone<br>↑ time to reach goal	-	-
[23]	♂Wi<br>From 4-5W<br>For 9W:<br>HFD 45% fat<br>(based on D12451, RD)<br>CON 11% fat	↑ BW	ETM<br><br>OFT	↑ latency to leave enclosed arm<br>↔ squares crossed	-	-

	(ns, Nuvilab)					
[24]	♂Wi<br>From 5-8W<br>For 9W:<br>HFD 45% fat<br>(ref. ns, PragSoluções Biociências)<br>CON 11% fat<br>(ref. ns, Nuvilab)	↑ BW<br>↑ adiposity	ETM<br><br>OFT	↑ latency to leave enclosed arm<br>↔ rearing, time in centre	HC<br>AMY	↑ IL6, ↔ TNFα<br>↔ IL6, ↑ TNFα
[25]	♂ SD<br>From 7-8 W<br>For 16W:<br>HFD 45% fat<br>CON 13.6% fat<br>(ref. ns, Wytwórnia Pasz Morawski Kcynia)	↑ BW	FST	↓ time immobile	FCx<br><br><br><br><br><br><br><br><br><br>HC	↔ G6P, Gly, GLUT1, GLUT4, PFK1L, Lac; G6PD, PDH activity<br>↑ INS, ↔ ⓅINSRβ/IINSRβ, ⓅAKT<br>↓ GLP-1, ↔ GLP1R, GLP2R<br>↑ ETC CIV-V, ↔ ETC CI-III<br>Δ OCR (↔ basal, ↔ ADP, ↓ FCCP, ↔ proton leak)<br>↔ ATP<br>↔ UCP4<br>↑ G6P<br>↔ Gly, GLUT1, GLUT4, PFK1L, Lac<br>↔ G6PD, ↓ PDH activity<br>↔ INS, ⓅINSRβ/INSRβ, ⓅAKT<br>↔ GLP-1, GLP1R, GLP2R<br>↔ ETC CI-V<br>↔ ATP<br>↔ UCP4
[26]	♂B6<br>From 3W<br>For 20W:<br>HFD 45% fat<br>(D12451, RD)<br>CON min. 4.5% fat<br>(ref. ns, Purina)		-	-	HYP	↑ POMC neurons with auto-phagosomes<br>Δ MT morphology<br>↓ number of POMC cells
[27]	♂B6<br>From 3-4W<br>For 32W:<br>HFD 45% fat<br>(D12451, RD)<br>CON 12% fat<br>(2016, Envigo)	↑ BW<br>↑ glucose	OFT<br>NORT	↔ total distance<br>↓ discrimination ratio	HC	↓ IDE<br>↔ PGC1α, PPARγ<br>↓ ETC CI-II, ↔ ETC CIII-V<br>↔ SOD, GPX<br>↑ HNE<br>↓ SOD1-immunoreactivity<br>↔ CS activity<br>↓ SYP, NRXN 2<br>↔ BDNF, SPL, DBN1, NLGN3<br>↓ number of dendritic spines<br>↑ GFAP immuno-reactivity<br>↑ IBA1 immuno-reactivity
	**HFD + psychostimulant drugs before puberty and during early adolescence**					
[28]	♂LE<br>From 3W<br>For 15-17W:<br>HFD 45% fat<br>(D12451, RD)<br>CON 8% fat<br>(A04, SAFE)	↑ BW<br>↑ insulin<br>↑ leptin<br>Δ lipids	AC	↔ locomotion<br>↔ acute AMPH-induced (1 mg/kg i.p.) locomotion<br>↑ AMPH-induced sensitization (2X 1 mg/kg i.p., 24h interval)	VTA<br><br><br><br><br>NAc<br><br><br><br><br>PFC	↔ spontaneous & busting activity of DA neurons<br>↑ AMPH-induced busting activity of DA neurons<br>↔ TH, DAT<br>↔ basal DA release<br>↔ TH, DAT, D1 D2<br>↑ AMPH-induced DA release and TH<br>↑ AMPH-induced c-Fos expression, D2R, ↔ D1R<br>↔ AMPH-induced c-Fos expression
[29]	♂B6<br>From 4W<br>For 4W:<br>HFD 45% fat<br>(D12451, RD	↑ BW<br>↑ adiposity<br>↑ leptin	CPP	↓ cocaine-induced CPP (3*2 mg/kg i.p.)<br>↔ cocaine-induced CPP<br>(3*4 or 8 mg/kg i.p.)	-	-

	CON 10% fat<br>(D12450B, RD)		PAT	↑ CPP for neutral food<br>↑ CPP for chocolate<br>↔ latency to aversive zone		
[30] *	♂♀OF1<br>From 6W<br>For 5 days<br>MWF, 2hr/day<br>HFD 45% fat<br>(TD.06415, Tekad)<br>CON 13% fat<br>(2014, Harlan Teklad)	↔ leptin<br>↔ ghrelin	CPP	↔ cocaine-induced CPP (4*10 mg/kg i.p.)<br>X RST of cocaine CPP<br>↔ EXT of cocaine CPP	STR	↑ mRNA *Oprμ* (♂)<br>↔ mRNA *Cbr*1
[31]	♂OF1<br>From 4W<br>For 5-6W:<br>HFD 45% fat<br>(TD.06415, Tekad)<br>CON 13% fat<br>(2014, Harlan Teklad)	↑ BW<br>↑ leptin<br>↓ ghrelin	CPP	↔ cocaine-induced CPP (4*1 or 6 mg/kg i.p.)<br>↔ EXT of cocaine CPP<br>↔ RST cocaine CPP	NAc<br>PFC<br>VTA	↓ mRNA *Cb1*, ↑ mRNA *Mtor*<br>↓ mRNA *Cb1*<br>↓ mRNA *Ghsr*
			EPM	↔ time in open arms		
			OFT	↔ cocaine-induced locomotion (10 mg/kg i.p.)		
	COC WD-2W<br>For 11-20W:<br>HFD 45% fat<br>(TD.06415, Tekad)<br>CON 13% fat<br>(2014, Harlan Teklad)		CPP	↑ EXT of cocaine CPP<br>↔ RST cocaine CPP		
	WD from HFD<br>For 2W:		EPM	↓ time in open arms<br>↑ cocaine-induced CPP (4*1 mg/kg i.p.)		
			CPP	↔ cocaine-induced CPP (4*6 mg/kg i.p.)<br>↔ EXT of cocaine CPP<br>↔ RST cocaine CPP	NAc<br>PFC<br>VTA	↓ *Cb1*, ↔ *Mtor*<br>↔ *Cb1*<br>↔ *Gshr*
			OFT	↔ cocaine-induced locomotion (10 mg/kg i.p.)		
[31] *	♂OF1<br>From 4W<br>For 5-6W:<br>MWF, 2hr/day<br>HFD 45% fat<br>(TD.06415, Tekad)<br>CON 13% fat<br>(2014, Harlan Teklad)	↔ BW<br>↔ leptin<br>↓ ghrelin<br>↔ CORT	CPP	↑ cocaine-induced CPP (4*1 mg/kg i.p.)<br>↔ cocaine-induced CPP (4*6 mg/kg i.p.)<br>↔ EXT of cocaine CPP<br>↔ RST of cocaine CPP		
			EPM	↓ time in open arms		
	WD from HFD<br>For2W		CPP	↔ cocaine-induced CPP<br>↓ EXT of cocaine CPP<br>↑ RST of cocaine CPP		
	♂CD1<br>From 4W<br>For 5-6W:<br>MWF, 2hr/day<br>HFD 45% fat<br>(TD.06415, Tekad)<br>CON 13% fat<br>(2014, Harlan Teklad)		SA	↑ cocaine-induced SA (0.5 mg/kg i.v.)<br>↑ RST of cocaine SA		

[32]<br>*<br>$	♂OF1<br>From 4W<br>For 17W:<br>MWF, 2hr/day<br>HFD 45% fat<br>(TD.06415, Tekad)<br>CON 13% fat<br>(2014, Harlan<br>Teklad)	↔ BW<br>↑ binge (SH)<br>↑ leptin (GH)<br>↓ CORT (SH)	CPP	↑ cocaine-induced<br>CPP (4*1 mg/kg i.p.)<br>(GH)<br>↓ cocaine-induced<br>CPP (4*1 mg/kg i.p.)<br>(SH)<br>↔ EXT of cocaine CPP<br>↔ RST of cocaine CPP		
[33]	♂Wi<br>From 7:8W<br>For 8:9W<br>HFD 50% fat<br>(modified AIN-93G,<br>Dyets)<br>CON 17% fat<br>(AIN-93G, Dyets)	↔BW<br>↔ leptin<br>↔ insulin<br>↔ glucose	CPP<br><br><br><br>AC	↓ AMPH -induced<br>CPP (1.5 mg/kg i.p.)<br>↔ AMPH-induced<br>CPP (3 mg/kg i.p.)<br>↓ AMPH-induced<br>sensitization (5X 1.5<br>mg/kg i.p., 10 days<br>interval, 0.75 mg/kg<br>i.p. challenge)	NAc	↓ DAT<br>↑ D1R, PDAPP 35 Thr75, Glur1<br>↔ D2R, PDAPP 35 Thr34<br>↔ DA, DA turnover, DOPAC
[34]	♂Wi<br>From 8-9W<br>For 5-6W:<br>HFD 50% fat<br>CON 30% fat<br>(ref. & supplier ns)<br>+ COC (15*15 mg/kg<br>i.p.)	↔ BW<br>Δ lipids	FST	↔ immobility<br>↔ climbing		
	First 5 days of COC<br>WD		FST	↓ immobility<br>↑ climbing		
			**HFD during late adolescence**			
[35]	♂B6<br>From 7W<br>For 3-6W:<br>HFD 45% fat<br>CON 10% fat<br>(ref. ns, RD)	↑ BW<br>↑ leptin<br>↑ insulin<br>↔ glucose<br>↔ CORT	SIT	↔ time exploring<br>mouse	-	-
[36]	♂W<br>From 8-10W<br>For 4W:<br>HFD 45% fat<br>CON 10% fat<br>(ref. & supplier ns)	-	EPM	↓ time in open arms	Blood	↓ GPX, GSH<br>↑ MDA
[37]	♂B6<br>From 6W<br>For 8W:<br>HFD 48% fat<br>(ref. & supplier ns)<br>CON 16% fat<br>(AIN-93G, NS)	↑ BW<br>↑ adiposity<br>↑ leptin<br>↑ glucose<br>Δ lipids	MBT	↑ % marbles buried	PFC	↑ mRNA *Drd2*<br>↔ mRNA *Drd1*<br>↓ mRNA *Gabbr1, Gabbr2*
[38]	♂B6<br>From 9W<br>For 8W:<br>HFD 45%<br>fat(D12451, RD)<br>CON 10% fat<br>(D12450B, RD)	↑ BW<br>↑ glucose<br>glucose<br>intolerance	TM	↓ spontaneous<br>alternation	FCx<br><br><br>HC	↔ ⓅAKT, ⓅGSK3β<br>X INS-stimulated ⓅAKT, ⓅGSK3β<br>↔ ⓅAKT<br>X INS-stimulated ⓅAKT
[39]	♂B6<br>From 6:7W<br>For 14W<br>HFD 42% fat<br>(TD.88137, Harlan)<br>CON 12% fat<br>(T2016S.15, Harlan)	↑ BW<br>↑ lipids	OFT<br>FST	↓ crossing events<br>↑ time immobile	HC<br><br><br><br><br>Cx	↓ Nissl staining CA1, CA3, ↔ DG<br>↓ mRNA *Bndf, Htr1a, Slc6a4, Ido2*, ↔<br>mRNA *Ido1*<br>↑ mRNA *Il1β, Il6, Il2, Il17*<br>↓ mRNA *Il10*<br>↔ mRNA *Il4, Tnfα, Tgfβ, Ifnγ*<br>↓ mRNA *Il1β, Il2, Il1*<br>↔ mRNA *Il6, Il10, Il4, Tnfα, Tgfβ, Ifnγ*

[40]	♂B6<br>From 8W<br>For 12 or 16W:<br>HFD 45% fat<br>(D12451, RD)<br>CON 8% fat<br>(A04, SAFE)	↑ BW<br>↑ glucose<br>↑ insulin<br>glucose<br>intolerance	OFT<br>NSF<br>TST	↓ centre time, entries<br>↑ latency to feed<br>↔ time immobile	vHC	↓ 5-HT level
[41]	♂B6<br>From 8W<br>For 5W:<br>HFD 45% fat<br>(D12451, RD)<br>CON 8% fat<br>(A04, SAFE)	-	-		HYP	↔ mRNA *Gfap*<br>↔ mRNA *Il1β*
[42]	♂B6<br>From 6-7W<br>For 20 or 40W:<br>HFD 45% fat<br>(D12451, RD)<br>CON 18% fat<br>(2018, Harlan<br>Teklad)	↑ BW<br>glucose<br>intolerance	MWM<br><br>OBT	↔ latency to find<br>platform<br>↓ ability to learn	HC	↔ INS-stimulated ⓅAKT/AKT<br>↔ CA1 LTP<br>↔ PSD95, SYP
[43]	♂CD1<br>From 7W<br>For 44W:<br>HFD 45% fat<br>(D12451, RD)<br>CON 13% fat<br>(ref. & supplier ns)	↑ BW<br>↓ glucose<br>↑ lipids	BM	↔ time to find escape<br>box	HC<br><br>HC/Cx	↔ CA1 pyramidal cell layer thickness<br>↔ GSH, GSSG, GSH/GSSG<br>↔ HNE<br>↔ NeuN, SYP, BDNF
[44]	♂B6<br>From 8W<br>For 5-38W:<br>HFD 45% fat<br>(D12451, RD)<br>CON 13% fat<br>(ref. ns, Oriental<br>Yeast)	↑ BW	DaLi<br>EPM<br>FST/TST<br>SPT<br>SxB<br>SIT<br><br>SRT<br><br>NORT	↔ time in light zone<br>↔ time in open arms<br>↔ time immobile<br>↓ sucrose preference<br>↓ mounting frequency<br>↑ time exploring<br>mouse<br>↓ social recognition<br>index<br>↔ discrimination<br>index	-	-
[45]	♀B6<br>From 9W<br>For 40W:<br>HFD 45% fat<br>(D12451, RD)<br>CON 10% fat<br>(D12450B, RD)	↑ BW gain<br>↑ adiposity<br>↑ leptin<br>↔ ADPN	EZM	↓ time in open areas		
				**Adult HFD**		
[46]	♂SD<br>From 10W<br>For 12W:<br>HFD 45% fat<br>(D12451, RD)<br>CON 10% fat<br>(D12450H, RD)	↑ BW<br>↑ leptin<br>↔ lipids	SPT	↓ sucrose preference	-	-
[47]	♂B6<br>From 10-11W<br>For 18W:<br>50% fat<br>(93075, Harlan<br>Teklad)<br>CON 10.6% fat<br>(4RF21, Mucedola)	↑ BW gain<br>↑ adiposity<br>↑ insulin<br>↑ glucose<br>↓ ADPN<br>↑ lipids	-	-	Serum<br>Cx	↑ TNFα, IL1β<br>↓ ACO activity<br>Δ OCR (↓ ADP-, ↓ FCCP, -↑ degree of<br>coupling)<br>↓ GSH, GSSG, GSH/GSSG<br>↓ SOD activity<br>↑ MDA<br>↓ BDNF, ↔ TrkB<br>↔ ⓅCREB/CREB

						↑ TNFα, IL1β
[48]	♂B6-<br>From 12W<br>For 24W:<br>45% fat<br>(D12451, RD)<br>CON 10% fat<br>(D12450B, RD)	↑ BW<br>↑ leptin<br>↑ insulin<br>↑ glucose<br>glucose intolerance<br>↔ CORT	OFT<br><br>YM	↓ crossing events, rearing<br>↓ spontaneous alternation	Cx	↓ PEA<br>↔ creatinine, Ⓟcreatine<br>↔ Gln, Glu, GABA, Glyc, Asp, MI, glucose, Lac<br>↔ GSH, As<br>↔ NAA<br>↔ SYP, SYN1, SNAP25, SYN4, PSD95, GPHN<br>↔ VGLUT1, VGLUT2 VGAT<br>↔ GFAP, IBA1
					HC	↔ PEA<br>↔ creatinine, Ⓟcreatine<br>↔ Gln, Glu, GABA, Glyc, Asp, MI, glucose, Lac<br>↔ GSH, As<br>↔ NAA<br>↓ SYN4, GPHN<br>↔ SYP, SYN1, SNAP25, PSD95<br>↓ VGLUT1, VGAT, ↔ VGLUT2<br>↔ GFAP, IBA1
					HYP	↔ PEA<br>↔ creatinine, Ⓟcreatine<br>↔ Gln, Glu, GABA, Glyc, Asp, MI, glucose, Lac<br>↔ GSH, As<br>↔ NAA<br>↔ SYP, SYN1, SNAP25, SYN4, PSD95, GPHN<br>↓ VGLUT1, VGLUT2 VGAT<br>↑ GFAP, ↔ IBA1
[49]	♂W<br>From 14W<br>For 16W:<br>HFD 45% fat<br>(D12451, RD)<br>CON 10% fat<br>(D12450, RD)		-	-	HYP	↑ *Insr, Obr*<br>↑ *Nr2b*<br>↑ *Tnfα, Il-1, Il-6*
[50]	♂B6<br>From 28-32W<br>For 4W:<br>HFD 45% fat<br>(D12452, RD)<br>CON 14% fat<br>(8604, Harlan Teklad)	↑ BW<br>↑ adiposity	OFT<br><br>TWAT	↔ time moving, time in centre<br>↔ shock avoidance, latency	HC	↑ mRNA *Oxr1*, ↔ *Oxr2*<br>↔ mRNA *Iba, Tnfα*
			**Adult HFD + psychostimulant**			
[51]	♂LE<br>From 10W<br>For 4-5W:<br>35.7% fat<br>(ref. & supplier ns)<br>CON 5.8% fat<br>(ref. ns, Harlan Teklad)	AMPH WD<br>↑ BW<br>↔ HFD intake<br>↑ chow intake	AC	↑ AMPH-induced locomotion (9*3 mg/kg i.p.)		
[52]	♂OF1<br>From 13W<br>For 7W:<br>HFD 40% fat<br>CON 30% fat<br>(ref. & supplier ns)<br>COC (5, 10, or 20 mg/kg i.p.)	↔ BW	OFT<br><br>EPM<br><br>FST	↓ cocaine-induced locomotion<br>↔ cocaine-induced anxiolytic effect<br>↑ cocaine-induced "antidepressant" effects		

Abbreviations

5-HT: serotonin; 5-HT1R: serotonin transporter; AC: activity cage; ACO: aconitase; ADP: adenosine diphosphate; ADPN, adiponectin; AMPH: amphetamine; AMY: amygdala; ARC: arcuate nucleus; As: ascorbate; Asp: aspartate; ATP: adenosine triphosphate; B6: C57BL6/J mice; BDNF/*Bdnf*: protein/gene coding for brain-derived neurotrophic factor; BM: Barnes maze test; BW: body weight; CA1: region 1 of the cornu Ammonis; CAT: catalase; *Cb1*: gene coding for cannabinoid receptor 1; CD1: CD1 mice; COC: cocaine; CORT: corticosterone; CON: control diet; CPP: conditioned place preference; CREB: cAMP-response element binding protein; *Crf*: gene coding for corticotropin-releasing factor; CS: citrate synthase; *Cytb*: gene coding for cytochrome b; DaLi: dark-light box test; DRD1/2 or *Drd1*/*drd2*: protein or gene coding for dopamine receptor 1 or 2; DA: dopamine; DAT: dopamine transporter; DBN1: debrin 1; DCF: dichlorodihydrofluorescein; DCX: doublecortin; DG: dentate gyrus; DIO: diet-induced obesity; DIR: diet-resistant obesity; DOPAC: 3,4-Dihydroxyphenylacetic acid; EPM, elevated plus-maze test; ETC CI-V: electron transport chain complex I-V; ETM: elevated T-maze; EXT, extinction; EZM: elevated zero-maze; FCx: frontal cortex; FCCP: trifluoromethoxy carbonylcyanide phenylhydrazone; FPT: fat preference test; FR: fixed ratio; FST: forced swim test; FUST: female urine sniffing test; G: gestation; G6P: glucose-6-phosphate; G6PD: glucose-6-phosphate dehydrogenase; GABA: gamma-aminobutyric acid; *Gabbr1*/*2*: gene coding for gamma-aminobutyric acid type B receptor sub-unit 1 or 2; GFAP/*Gfap*: protein/gene coding for glial fibrillary acidic protein; GH: group-housed; *Ghsr*: gene coding for growth hormone secretagogue receptor; Gln: glutamine; GLP1:glucagon-like peptide-1; GLP1/2R: glucagon-like peptide-1 or 2 receptor; Glu: glutamate; Glur1: glutamate ionotropic receptor AMPA type subunit 1; GLUT1/4: glucose transporter 1 or 4; Gly: glycogene; Glyc: glycine; GPHN: gephyrin; GPX: glutathione peroxidase; GSH: glutathione; GSK3β: glycogen synthase kinase 3 beta; GSSG: oxidised glutathione; HBT: hole board test; HC: hippocampus; HFD: high-fat diet; HNE: hydroxynonenal; *Htr1a*: gene coding for 5-hydroxytryptamine receptor 1A; HWM: Hebbs Williams maze test; HYP: hypothalamus; IBA1/*Iba1*: protein/gene coding for ionised calcium binding adaptor molecule 1; IDE: Insulin-Degrading Enzyme; *Ido1*/*2*: gene coding for indoleamine 2,3-dioxygenase; IFNγ/*Ifnγ*: protein/gene coding for interferon gamma; IL/Il: protein/gene coding for interleukin; INS: insulin; INSR/*Insr*: protein/gene coding for insulin receptor; *Irs1*: gene coding for insulin receptor substrate 1; Lac: lactate; LE: Long-Evans rats; LPS: lipopolysaccharide; LTP: long-term potentiation; MB: maternal behaviour; MBT: marble burying test; MC-4R: melanocortin receptor 4; MDA: malondialdehyde; MI: myoinositol; MMP: mitochondria membrane potential; MT: mitochondria; *Mtor*: gene coding for mechanistic target of rapamycin kinase; MWM: Morris water maze test; NAA: N-acetyl aspartate; NAc: nucleus accumbens; *Nd*: gene coding for NADH-ubiquinone oxidoreductase chain; NLGN3: neuroligin; NORT: novel object recognition test; *Nr2b*: gene coding for N-methyl-D-aspartate receptor subunit 2b; *Nr3c1*: gene coding for nuclear receptor subfamily 3 group C member 1 (glucocorticoid Receptor); *Nr3c2*: gene coding for nuclear receptor subfamily 3 group C member 2 (mineralocorticoid Receptor); NRX2: neurexin 2; ns: non specified; NSF. novelty-suppressed feeding test; *Obr*: gene coding for leptin receptor; OBT: operant bar-pressing task; OCR: oxygen consumption rate; OFT: open-field test; OF1: OF1 mice; *Oprμ*: gene coding for opioid mu receptor; *Oxr*; gene coding for orexin receptor; PAT: passive avoidance task; PDH: pyruvate dehydrogenase; PEA: phosphorylethanolamine; PFC: prefrontal cortex; PFK1L: phosphofructokinase 1; PGC1α: peroxisome proliferator-activated receptor gamma coactivator 1-alpha; PND: post-natal day; POMC: pro-opiomelanocortin; PPARγ: peroxisome proliferator-activated receptor gamma; PR: progressive ratio; PSD95: postsynaptic density protein 95; PVN: paraventricular nucleus of the hypothalamus; RAM: radial arm maze test; RD: Research Diets, Inc.; RW: running wheel; SH: single-housed; *Slc6a4*: gene coding for sodium-dependent serotonin transporter; RST: reinstatement; SA: self-administration; SD: Sprague-Dawley; SIT: social interaction test; SNAP25: synaptosomal-associated protein 25; SOD: superoxide dismutase; SPL: spinophilin; SPT: sucrose preference test; SRT: social recognition test; STR: striatum; SxB, sexual behaviour; SYN1/4: syntaxin1 or 4; SYP: synaptophysin; TAS: total antioxidant status; TGFβ/*Tgfβ*: protein/gene coding for transforming growth factor beta; TH: tyrosine hydroxylase; TM: T-maze test; TNFα/*Tnfα*: protein/gene coding for tumour necrosis factor; TrkB: tropomyosin receptor kinase B; TST: tail suspension test; TWAT: two-way active avoidance task; UCP4, uncoupling protein 4; VGAT: vesicular GABA transporter; VGLUT1/2: vesicular glutamate transporter 1 or 2; vHC: ventral hippocampus; VTA: ventral tegmental area; W: week; WD: withdrawal; Wi: Wistar rats; WH: Wistar Han rats; YM: Y-maze test.

Symbols

* limited access to a high-fat diet; $: outcomes differing based on the housing conditions;Ⓟ phosphorylated; ↑: significant increase; ↓: significant decrease; ↔: no significant changes; Δ: significant changes.

Supplementary Table S2. Effects of cocaine on behaviour and brain.

The column "Design" refers to the rodent sex, strain, species and age. It also indicates the dose, route and pattern of psychostimulant (cocaine, COC) drug injections. Nomenclature for genes (lower cases, italicized) and proteins (upper cases) is respected in the table.

Ref	Design.	Test	Findings	Tissue sampled	Findings
[53]	♂ B6-8:12W<br>♂ SD-6:7W<br>COC (7*20 mg/kg i.p. or 10*1 mg/kg i.v.)	-	-	NAc	↑ Egr3 binding to promoters of mitochondrial-related genes (*Drp1*, *Nrf2*, *Polγ*)<br>↑ mRNA *Nrf1*, *Nrf2*, *Tomm20*<br>↓ mRNA *Drp1*, *Polγ*, *Tfam*<br>Δ gene expression in D1- vs D2-MSNs
[54]	♂ B6-age ns				
	COC (2*7.5 mg/kg i.p.)	CPP	↑ cocaine-induced CPP by OX of PCG1α in D1-MSNs (↓ in D2-MSNs)		
	COC (5*10 mg/kg i.p.)	OFT	↑ cocaine-induced locomotion by OX of PCG1α in D1-MSNs (↔ in D2-MSNs)		
	COC (7*20 mg/kg i.p.)			NAc	↑ PGC1α/ mRNA *Pgc1a* in D1-MSNs<br>↓ PGC1α/ mRNA *Pgc1a* in D2-MSNs<br>↑ Egr3 binding to *Pgc1a* promoter
[55]	♂ B6-age ns				
	COC (2*7.5 mg/kg i.p.)	CPP	X cocaine-induced CPP by inhibition of mitochondrial fission		
	COC (7*10 + 10 mg/kg i.p)	OFT	X cocaine-induced locomotion by inhibition of mitochondrial fission	NAc	↑ c-Fos, AMPA/NMDA ratio and rectification index in D1-MSNs
	COC (10*1 mg/kg i.v.)	SA	X cocaine SA by inhibition of mitochondrial fission		↑ mRNA *Drp1*, *Mfn2*<br>↔ mRNA *Fis1*, *Mfn1*, *Opa1*<br>↓ mitochondria size in D1-MSNs
	COC (7*20 mg/kg i.p.)	-			↑ mRNA *Drp1* in D1-MSNs<br>↓ mRNA *Drp1* in D2-MSNs<br>↔ mRNA *Mfn1*, *Mfn2*, *Opa1*
[56]	♂Wi-5:6W<br>COC (3*20 mg/kg i.p.)	CPP	cocaine-induced CPP	NAc	Δ metabolites<br>↑ NAA, lactate, Glu, succinate, GABA, creatine, taurine<br>↓ leucine, 3-hydroxybutyric acid, L-lysine, cysteine, myoinositol
				STR	Δ metabolites<br>↑ NAA, GABA, choline, taurine<br>↓ 3-hydroxybutyric acid, lactate, creatine, glycerol, myoinositol
[57]	♂Wi-6:7W<br>COC (20 mg/kg i.p.)	-	-	Cg	↓ mRNA coding for ND1, ND2, ND4, ND5, COX2
				STR	↔ mRNA coding for ND1, ND2, ND4, ND5, COX2
				CB	↔ mRNA coding for ND1, ND2, ND4, ND5, COX2
[58]	♂Wi-6:8W<br>COC (7*15 mg/kg i.p.)	-	-	WB	↑ nNOS activity<br>↑ MDA<br>↓ GSH<br>↑ SOD, GPX activity<br>↓ CAT, GR, GST activity

					↓ GSH (synaptosomal mitochondria)
[59]	♂Wi-7:8W COC (12*0.5 mg/kg i.v.)	-	-	PFC/HC	↑ mtDNA cn<br>↑ mRNA coding for ND1, ND6<br>↑ mRNA *Ndufaf2, Uqcrq, Oxnad1, Cox7c, Tfam, Mfn1, Opa1, Mtfr1, and Opa3*

<u>Abbreviations</u>

AMPA/NMDA: α-amino-3-hydroxy-5-methyl-4-isoxazolepropionic acid receptor/ N-methyl-D-aspartate receptor; B6, C57BL6/J mice; CAT: catalase; COC: cocaine; COX2: gene coding for mitochondrially encoded cytochrome C oxidase II; *Cox7c*: Cytochrome C Oxidase Subunit 7C; CPP, conditioned place preference; D1-MSNs: dopamine receptor D1-expressing medium spiny neurons; D2-MSNs: dopamine receptor D2-expressing medium spiny neurons; *Drp1:* gene coding for Dynamin 1 Like*; Egr3*: gene coding for Early Growth Response 3; *Fis1*: gene coding for mitochondrial fission 1; GABA: gamma-aminobutyric acid; Glu: glutamate; GPX: glutathione peroxidase; GR: glutathione reductase; GSH: glutathione; GST: Glutathione S-transferase; i.p.: intraperitoneal; i.v.: intravenous; MDA: malondialdehyde; *Mfn1*/*2*: gene coding for mitofusin 1 or 2; mtDNA cn: mitochondrial DNA copy number; NAA: N-acetyl aspartate; NAc: nucleus accumbens; ND/Nd: NADH-ubiquinone oxidoreductase chain; *Ndufaf*2: NADH: Ubiquinone Oxidoreductase Complex Assembly Factor 2; *Nrf1/2:* gene coding for Nuclear Respiratory Factor 1 or 2; ns: not specified*;* nNos: Neuronal Nitric Oxide Synthase; OFT: open-field; *Opa1:* Mitochondrial Dynamin Like GTPase; OX: overexpression; OXNAD1: Oxidoreductase NAD Binding Domain Containing 1; PGC1α/*Pgc1α*: protein/gene coding for peroxisome proliferator-activated receptor gamma coactivator 1-alpha; Polγ*:* gene coding for *DNA polymerase gamma,* RCR: respiratory coupling ratio; SA, self-administration; SD, Sprague-Dawley; SOD: superoxide dismutase; STR: striatum; *Tfam*: gene coding for Transcription Factor A; *Tomm20*: translocase of outer mitochondrial membrane 20; *Uqcrq*: Ubiquinol-Cytochrome C Reductase Complex III Subunit VII; W, week; WD, withdrawal; Wi, Wistar rats.

<u>Symbols</u>

↑: significant increase; ↓: significant decrease; ↔: no significant changes; X: blockade; Δ: significant changes.

References

1. Rodriguez, J.S.; Rodríguez-González, G.L.; Reyes-Castro, L.A.; Ibáñez, C.; Ramírez, A.; Chavira, R.; Larrea, F.; Nathanielsz, P.W.; Zambrano, E. Maternal Obesity in the Rat Programs Male Offspring Exploratory, Learning and Motivation Behavior: Prevention by Dietary Intervention Pre-Gestation or in Gestation. *Int J Dev Neurosci* **2012**, *30*, 75–81, doi:10.1016/j.ijdevneu.2011.12.012.
2. Leuthardt, A.S.; Bayer, J.; Monné Rodríguez, J.M.; Boyle, C.N. Influence of High Energy Diet and Polygenic Predisposition for Obesity on Postpartum Health in Rat Dams. *Front Physiol* **2021**, *12*, 772707, doi:10.3389/fphys.2021.772707.
3. Baptissart, M.; Lamb, H.E.; To, K.; Bradish, C.; Tehrani, J.; Reif, D.; Cowley, M. Neonatal Mice Exposed to a High-Fat Diet in Utero Influence the Behaviour of Their Nursing Dam. *Proc Biol Sci* **2018**, *285*, 20181237, doi:10.1098/rspb.2018.1237.
4. Connor, K.L.; Vickers, M.H.; Beltrand, J.; Meaney, M.J.; Sloboda, D.M. Nature, Nurture or Nutrition? Impact of Maternal Nutrition on Maternal Care, Offspring Development and Reproductive Function. *J Physiol* **2012**, *590*, 2167–2180, doi:10.1113/jphysiol.2011.223305.
5. D'Asti, E.; Long, H.; Tremblay-Mercier, J.; Grajzer, M.; Cunnane, S.C.; Di Marzo, V.; Walker, C.-D. Maternal Dietary Fat Determines Metabolic Profile and the Magnitude of Endocannabinoid Inhibition of the Stress Response in Neonatal Rat Offspring. *Endocrinology* **2010**, *151*, 1685–1694, doi:10.1210/en.2009-1092.
6. Cunha, F. da S.; Dalle Molle, R.; Portella, A.K.; Benetti, C. da S.; Noschang, C.; Goldani, M.Z.; Silveira, P.P. Both Food Restriction and High-Fat Diet during Gestation Induce Low Birth Weight and Altered Physical Activity in Adult Rat Offspring: The "Similarities in the Inequalities" Model. *PLoS One* **2015**, *10*, e0118586, doi:10.1371/journal.pone.0118586.
7. Paradis, J.; Boureau, P.; Moyon, T.; Nicklaus, S.; Parnet, P.; Paillé, V. Perinatal Western Diet Consumption Leads to Profound Plasticity and GABAergic Phenotype Changes within Hypothalamus and Reward Pathway from Birth to Sexual Maturity in Rat. *Front Endocrinol (Lausanne)* **2017**, *8*, 216, doi:10.3389/fendo.2017.00216.
8. Gawlińska, K.; Gawliński, D.; Korostyński, M.; Borczyk, M.; Frankowska, M.; Piechota, M.; Filip, M.; Przegaliński, E. Maternal Dietary Patterns Are Associated with Susceptibility to a Depressive-like Phenotype in Rat Offspring. *Dev Cogn Neurosci* **2021**, *47*, 100879, doi:10.1016/j.dcn.2020.100879.
9. Naef, L.; Srivastava, L.; Gratton, A.; Hendrickson, H.; Owens, S.M.; Walker, C.-D. Maternal High Fat Diet during the Perinatal Period Alters Mesocorticolimbic Dopamine in the Adult Rat Offspring: Reduction in the Behavioral Responses to Repeated Amphetamine Administration. *Psychopharmacology (Berl)* **2008**, *197*, 83–94, doi:10.1007/s00213-007-1008-4.
10. Gawliński, D.; Gawlińska, K.; Frankowska, M.; Filip, M. Maternal Diet Influences the Reinstatement of Cocaine-Seeking Behavior and the Expression of Melanocortin-4 Receptors in Female Offspring of Rats. *Nutrients* **2020**, *12*, E1462, doi:10.3390/nu12051462.
11. Janthakhin, Y.; Rincel, M.; Costa, A.-M.; Darnaudéry, M.; Ferreira, G. Maternal High-Fat Diet Leads to Hippocampal and Amygdala Dendritic Remodeling in Adult Male Offspring. *Psychoneuroendocrinology* **2017**, *83*, 49–57, doi:10.1016/j.psyneuen.2017.05.003.
12. Niu, X.; Wu, X.; Ying, A.; Shao, B.; Li, X.; Zhang, W.; Lin, C.; Lin, Y. Maternal High Fat Diet Programs Hypothalamic-Pituitary-Adrenal Function in Adult Rat Offspring. *Psychoneuroendocrinology* **2019**, *102*, 128–138, doi:10.1016/j.psyneuen.2018.12.003.
13. Lépinay, A.L.; Larrieu, T.; Joffre, C.; Acar, N.; Gárate, I.; Castanon, N.; Ferreira, G.; Langelier, B.; Guesnet, P.; Brétillon, L.; et al. Perinatal High-Fat Diet Increases

Hippocampal Vulnerability to the Adverse Effects of Subsequent High-Fat Feeding. *Psychoneuroendocrinology* **2015**, *53*, 82–93, doi:10.1016/j.psyneuen.2014.12.008.
14. Toniazzo, A.P.; Arcego, D.M.; Lazzaretti, C.; Mota, C.; Schnorr, C.E.; Pettenuzzo, L.F.; Krolow, R.; Fonseca Moreira, J.C.; Dalmaz, C. Sex-Dependent Effect on Mitochondrial and Oxidative Stress Parameters in the Hypothalamus Induced by Prepubertal Stress and Access to High Fat Diet. *Neurochem Int* **2019**, *124*, 114–122, doi:10.1016/j.neuint.2019.01.008.
15. White, K.A.; Hutton, S.R.; Weimer, J.M.; Sheridan, P.A. Diet-Induced Obesity Prolongs Neuroinflammation and Recruits CCR2(+) Monocytes to the Brain Following Herpes Simplex Virus (HSV)-1 Latency in Mice. *Brain Behav Immun* **2016**, *57*, 68–78, doi:10.1016/j.bbi.2016.06.007.
16. Terrien, J.; Seugnet, I.; Seffou, B.; Herrero, M.J.; Bowers, J.; Chamas, L.; Decherf, S.; Duvernois-Berthet, E.; Djediat, C.; Ducos, B.; et al. Reduced Central and Peripheral Inflammatory Responses and Increased Mitochondrial Activity Contribute to Diet-Induced Obesity Resistance in WSB/EiJ Mice. *Sci Rep* **2019**, *9*, 19696, doi:10.1038/s41598-019-56051-4.
17. Del Rio, D.; Morales, L.; Ruiz-Gayo, M.; Del Olmo, N. Effect of High-Fat Diets on Mood and Learning Performance in Adolescent Mice. *Behav Brain Res* **2016**, *311*, 167–172, doi:10.1016/j.bbr.2016.04.052.
18. Del Rosario, A.; McDermott, M.M.; Panee, J. Effects of a High-Fat Diet and Bamboo Extract Supplement on Anxiety- and Depression-like Neurobehaviours in Mice. *Br J Nutr* **2012**, *108*, 1143–1149, doi:10.1017/S0007114511006738.
19. Valladolid-Acebes, I.; Stucchi, P.; Cano, V.; Fernández-Alfonso, M.S.; Merino, B.; Gil-Ortega, M.; Fole, A.; Morales, L.; Ruiz-Gayo, M.; Del Olmo, N. High-Fat Diets Impair Spatial Learning in the Radial-Arm Maze in Mice. *Neurobiol Learn Mem* **2011**, *95*, 80–85, doi:10.1016/j.nlm.2010.11.007.
20. Aslani, S.; Vieira, N.; Marques, F.; Costa, P.S.; Sousa, N.; Palha, J.A. The Effect of High-Fat Diet on Rat's Mood, Feeding Behavior and Response to Stress. *Transl Psychiatry* **2015**, *5*, e684, doi:10.1038/tp.2015.178.
21. Finger, B.C.; Dinan, T.G.; Cryan, J.F. High-Fat Diet Selectively Protects against the Effects of Chronic Social Stress in the Mouse. *Neuroscience* **2011**, *192*, 351–360, doi:10.1016/j.neuroscience.2011.06.072.
22. Blanco-Gandía, M.C.; Miñarro, J.; Rodríguez-Arias, M. Behavioral Profile of Intermittent vs Continuous Access to a High Fat Diet during Adolescence. *Behav Brain Res* **2019**, *368*, 111891, doi:10.1016/j.bbr.2019.04.005.
23. de Noronha, S.R.; Campos, G.V.; Abreu, A.R.; de Souza, A.A.; Chianca, D.A.; de Menezes, R.C. High Fat Diet Induced-Obesity Facilitates Anxiety-like Behaviors Due to GABAergic Impairment within the Dorsomedial Hypothalamus in Rats. *Behav Brain Res* **2017**, *316*, 38–46, doi:10.1016/j.bbr.2016.08.042.
24. Noronha, S.S.R.; Lima, P.M.; Campos, G.S.V.; Chírico, M.T.T.; Abreu, A.R.; Figueiredo, A.B.; Silva, F.C.S.; Chianca, D.A.; Lowry, C.A.; De Menezes, R.C.A. Association of High-Fat Diet with Neuroinflammation, Anxiety-like Defensive Behavioral Responses, and Altered Thermoregulatory Responses in Male Rats. *Brain Behav Immun* **2019**, *80*, 500–511, doi:10.1016/j.bbi.2019.04.030.
25. Głombik, K.; Detka, J.; Góralska, J.; Kurek, A.; Solnica, B.; Budziszewska, B. Brain Metabolic Alterations in Rats Showing Depression-Like and Obesity Phenotypes. *Neurotox Res* **2020**, *37*, 406–424, doi:10.1007/s12640-019-00131-w.
26. Thaler, J.P.; Yi, C.-X.; Schur, E.A.; Guyenet, S.J.; Hwang, B.H.; Dietrich, M.O.; Zhao, X.; Sarruf, D.A.; Izgur, V.; Maravilla, K.R.; et al. Obesity Is Associated with Hypothalamic Injury in Rodents and Humans. *J Clin Invest* **2012**, *122*, 153–162, doi:10.1172/JCI59660.

27. Busquets, O.; Ettcheto, M.; Eritja, À.; Espinosa-Jiménez, T.; Verdaguer, E.; Olloquequi, J.; Beas-Zarate, C.; Castro-Torres, R.D.; Casadesús, G.; Auladell, C.; et al. C-Jun N-Terminal Kinase 1 Ablation Protects against Metabolic-Induced Hippocampal Cognitive Impairments. *J Mol Med (Berl)* **2019**, *97*, 1723–1733, doi:10.1007/s00109-019-01856-z.
28. Naneix, F.; Tantot, F.; Glangetas, C.; Kaufling, J.; Janthakhin, Y.; Boitard, C.; De Smedt-Peyrusse, V.; Pape, J.R.; Vancassel, S.; Trifilieff, P.; et al. Impact of Early Consumption of High-Fat Diet on the Mesolimbic Dopaminergic System. *eNeuro* **2017**, *4*, ENEURO.0120-17.2017, doi:10.1523/ENEURO.0120-17.2017.
29. Morales, L.; Del Olmo, N.; Valladolid-Acebes, I.; Fole, A.; Cano, V.; Merino, B.; Stucchi, P.; Ruggieri, D.; López, L.; Alguacil, L.F.; et al. Shift of Circadian Feeding Pattern by High-Fat Diets Is Coincident with Reward Deficits in Obese Mice. *PLoS One* **2012**, *7*, e36139, doi:10.1371/journal.pone.0036139.
30. Ródenas-González, F.; Blanco-Gandía, M.D.C.; Pascual, M.; Molari, I.; Guerri, C.; López, J.M.; Rodríguez-Arias, M. A Limited and Intermittent Access to a High-Fat Diet Modulates the Effects of Cocaine-Induced Reinstatement in the Conditioned Place Preference in Male and Female Mice. *Psychopharmacology (Berl)* **2021**, *238*, 2091–2103, doi:10.1007/s00213-021-05834-7.
31. Blanco-Gandía, M.C.; Cantacorps, L.; Aracil-Fernández, A.; Montagud-Romero, S.; Aguilar, M.A.; Manzanares, J.; Valverde, O.; Miñarro, J.; Rodríguez-Arias, M. Effects of Bingeing on Fat during Adolescence on the Reinforcing Effects of Cocaine in Adult Male Mice. *Neuropharmacology* **2017**, *113*, 31–44, doi:10.1016/j.neuropharm.2016.09.020.
32. Blanco-Gandía, M.C.; Montagud-Romero, S.; Aguilar, M.A.; Miñarro, J.; Rodríguez-Arias, M. Housing Conditions Modulate the Reinforcing Properties of Cocaine in Adolescent Mice That Binge on Fat. *Physiol Behav* **2018**, *183*, 18–26, doi:10.1016/j.physbeh.2017.10.014.
33. Hryhorczuk, C.; Florea, M.; Rodaros, D.; Poirier, I.; Daneault, C.; Des Rosiers, C.; Arvanitogiannis, A.; Alquier, T.; Fulton, S. Dampened Mesolimbic Dopamine Function and Signaling by Saturated but Not Monounsaturated Dietary Lipids. *Neuropsychopharmacology* **2016**, *41*, 811–821, doi:10.1038/npp.2015.207.
34. Loebens, M.; Barros, H.M.T. Diet Influences Cocaine Withdrawal Behaviors in the Forced Swimming Test. *Pharmacol Biochem Behav* **2003**, *74*, 259–267, doi:10.1016/s0091-3057(02)00924-3.
35. Finger, B.C.; Dinan, T.G.; Cryan, J.F. The Temporal Impact of Chronic Intermittent Psychosocial Stress on High-Fat Diet-Induced Alterations in Body Weight. *Psychoneuroendocrinology* **2012**, *37*, 729–741, doi:10.1016/j.psyneuen.2011.06.015.
36. Ganji, A.; Salehi, I.; Sarihi, A.; Shahidi, S.; Komaki, A. Effects of Hypericum Scabrum Extract on Anxiety and Oxidative Stress Biomarkers in Rats Fed a Long-Term High-Fat Diet. *Metab Brain Dis* **2017**, *32*, 503–511, doi:10.1007/s11011-016-9940-9.
37. Martins de Carvalho, L.; Lauar Gonçalves, J.; Sondertoft Braga Pedersen, A.; Damasceno, S.; Elias Moreira Júnior, R.; Uceli Maioli, T.; Faria, A.M.C. de; Brunialti Godard, A.L. High-Fat Diet Withdrawal Modifies Alcohol Preference and Transcription of Dopaminergic and GABAergic Receptors. *J Neurogenet* **2019**, *33*, 10–20, doi:10.1080/01677063.2018.1526934.
38. Arnold, S.E.; Lucki, I.; Brookshire, B.R.; Carlson, G.C.; Browne, C.A.; Kazi, H.; Bang, S.; Choi, B.-R.; Chen, Y.; McMullen, M.F.; et al. High Fat Diet Produces Brain Insulin Resistance, Synaptodendritic Abnormalities and Altered Behavior in Mice. *Neurobiol Dis* **2014**, *67*, 79–87, doi:10.1016/j.nbd.2014.03.011.
39. Wu, H.; Lv, W.; Pan, Q.; Kalavagunta, P.K.; Liu, Q.; Qin, G.; Cai, M.; Zhou, L.; Wang, T.; Xia, Z.; et al. Simvastatin Therapy in Adolescent Mice Attenuates HFD-Induced Depression-like Behavior by Reducing Hippocampal Neuroinflammation. *J Affect Disord* **2019**, *243*, 83–95, doi:10.1016/j.jad.2018.09.022.

40. Zemdegs, J.; Quesseveur, G.; Jarriault, D.; Pénicaud, L.; Fioramonti, X.; Guiard, B.P. High-Fat Diet-Induced Metabolic Disorders Impairs 5-HT Function and Anxiety-like Behavior in Mice. *Br J Pharmacol* **2016**, *173*, 2095–2110, doi:10.1111/bph.13343.
41. Lorenzo, P.I.; Martin Vazquez, E.; López-Noriega, L.; Fuente-Martín, E.; Mellado-Gil, J.M.; Franco, J.M.; Cobo-Vuilleumier, N.; Guerrero Martínez, J.A.; Romero-Zerbo, S.Y.; Perez-Cabello, J.A.; et al. The Metabesity Factor HMG20A Potentiates Astrocyte Survival and Reactive Astrogliosis Preserving Neuronal Integrity. *Theranostics* **2021**, *11*, 6983–7004, doi:10.7150/thno.57237.
42. Mielke, J.G.; Nicolitch, K.; Avellaneda, V.; Earlam, K.; Ahuja, T.; Mealing, G.; Messier, C. Longitudinal Study of the Effects of a High-Fat Diet on Glucose Regulation, Hippocampal Function, and Cerebral Insulin Sensitivity in C57BL/6 Mice. *Behav Brain Res* **2006**, *175*, 374–382, doi:10.1016/j.bbr.2006.09.010.
43. Li, L.; Wang, Z.; Zuo, Z. Chronic Intermittent Fasting Improves Cognitive Functions and Brain Structures in Mice. *PLoS One* **2013**, *8*, e66069, doi:10.1371/journal.pone.0066069.
44. Takase, K.; Tsuneoka, Y.; Oda, S.; Kuroda, M.; Funato, H. High-Fat Diet Feeding Alters Olfactory-, Social-, and Reward-Related Behaviors of Mice Independent of Obesity. *Obesity (Silver Spring)* **2016**, *24*, 886–894, doi:10.1002/oby.21441.
45. Griffin, T.M.; Fermor, B.; Huebner, J.L.; Kraus, V.B.; Rodriguiz, R.M.; Wetsel, W.C.; Cao, L.; Setton, L.A.; Guilak, F. Diet-Induced Obesity Differentially Regulates Behavioral, Biomechanical, and Molecular Risk Factors for Osteoarthritis in Mice. *Arthritis Res Ther* **2010**, *12*, R130, doi:10.1186/ar3068.
46. Hersey, M.; Woodruff, J.L.; Maxwell, N.; Sadek, A.T.; Bykalo, M.K.; Bain, I.; Grillo, C.A.; Piroli, G.G.; Hashemi, P.; Reagan, L.P. High-Fat Diet Induces Neuroinflammation and Reduces the Serotonergic Response to Escitalopram in the Hippocampus of Obese Rats. *Brain Behav Immun* **2021**, *96*, 63–72, doi:10.1016/j.bbi.2021.05.010.
47. Cavaliere, G.; Trinchese, G.; Penna, E.; Cimmino, F.; Pirozzi, C.; Lama, A.; Annunziata, C.; Catapano, A.; Mattace Raso, G.; Meli, R.; et al. High-Fat Diet Induces Neuroinflammation and Mitochondrial Impairment in Mice Cerebral Cortex and Synaptic Fraction. *Front Cell Neurosci* **2019**, *13*, 509, doi:10.3389/fncel.2019.00509.
48. Lizarbe, B.; Soares, A.F.; Larsson, S.; Duarte, J.M.N. Neurochemical Modifications in the Hippocampus, Cortex and Hypothalamus of Mice Exposed to Long-Term High-Fat Diet. *Front Neurosci* **2018**, *12*, 985, doi:10.3389/fnins.2018.00985.
49. De Souza, C.T.; Araujo, E.P.; Bordin, S.; Ashimine, R.; Zollner, R.L.; Boschero, A.C.; Saad, M.J.A.; Velloso, L.A. Consumption of a Fat-Rich Diet Activates a Proinflammatory Response and Induces Insulin Resistance in the Hypothalamus. *Endocrinology* **2005**, *146*, 4192–4199, doi:10.1210/en.2004-1520.
50. Duffy, C.M.; Hofmeister, J.J.; Nixon, J.P.; Butterick, T.A. High Fat Diet Increases Cognitive Decline and Neuroinflammation in a Model of Orexin Loss. *Neurobiol Learn Mem* **2019**, *157*, 41–47, doi:10.1016/j.nlm.2018.11.008.
51. Orsini, C.A.; Ginton, G.; Shimp, K.G.; Avena, N.M.; Gold, M.S.; Setlow, B. Food Consumption and Weight Gain after Cessation of Chronic Amphetamine Administration. *Appetite* **2014**, *78*, 76–80, doi:10.1016/j.appet.2014.03.013.
52. Erhardt, E.; Zibetti, L.C.E.; Godinho, J.M.; Bacchieri, B.; Barros, H.M.T. Behavioral Changes Induced by Cocaine in Mice Are Modified by a Hyperlipidic Diet or Recombinant Leptin. *Braz J Med Biol Res* **2006**, *39*, 1625–1635, doi:10.1590/s0100-879x2006001200014.
53. Cole, S.L.; Chandra, R.; Harris, M.; Patel, I.; Wang, T.; Kim, H.; Jensen, L.; Russo, S.J.; Turecki, G.; Gancarz-Kausch, A.M.; et al. Cocaine-Induced Neuron Subtype Mitochondrial Dynamics through Egr3 Transcriptional Regulation. *Mol Brain* **2021**, *14*, 101, doi:10.1186/s13041-021-00800-y.

54. Chandra, R.; Engeln, M.; Francis, T.C.; Konkalmatt, P.; Patel, D.; Lobo, M.K. A Role for Peroxisome Proliferator-Activated Receptor Gamma Coactivator-1α in Nucleus Accumbens Neuron Subtypes in Cocaine Action. *Biol Psychiatry* **2017**, *81*, 564–572, doi:10.1016/j.biopsych.2016.10.024.
55. Chandra, R.; Engeln, M.; Schiefer, C.; Patton, M.H.; Martin, J.A.; Werner, C.T.; Riggs, L.M.; Francis, T.C.; McGlincy, M.; Evans, B.; et al. Drp1 Mitochondrial Fission in D1 Neurons Mediates Behavioral and Cellular Plasticity during Early Cocaine Abstinence. *Neuron* **2017**, *96*, 1327-1341.e6, doi:10.1016/j.neuron.2017.11.037.
56. Li, Y.; Yan, G.-Y.; Zhou, J.-Q.; Bu, Q.; Deng, P.-C.; Yang, Y.-Z.; Lv, L.; Deng, Y.; Zhao, J.-X.; Shao, X.; et al. ^{1}H NMR-Based Metabonomics in Brain Nucleus Accumbens and Striatum Following Repeated Cocaine Treatment in Rats. *Neuroscience* **2012**, *218*, 196–205, doi:10.1016/j.neuroscience.2012.05.019.
57. Dietrich, J.-B.; Poirier, R.; Aunis, D.; Zwiller, J. Cocaine Downregulates the Expression of the Mitochondrial Genome in Rat Brain. *Ann N Y Acad Sci* **2004**, *1025*, 345–350, doi:10.1196/annals.1316.042.
58. Vitcheva, V.; Simeonova, R.; Kondeva-Burdina, M.; Mitcheva, M. Selective Nitric Oxide Synthase Inhibitor 7-Nitroindazole Protects against Cocaine-Induced Oxidative Stress in Rat Brain. *Oxid Med Cell Longev* **2015**, *2015*, 157876, doi:10.1155/2015/157876.
59. Sadakierska-Chudy, A.; Kotarska, A.; Frankowska, M.; Jastrzębska, J.; Wydra, K.; Miszkiel, J.; Przegaliński, E.; Filip, M. The Alterations in Mitochondrial DNA Copy Number and Nuclear-Encoded Mitochondrial Genes in Rat Brain Structures after Cocaine Self-Administration. *Mol Neurobiol* **2017**, *54*, 7460–7470, doi:10.1007/s12035-016-0153-3.

---

[i] Present address: Department of Behavioural and Molecular Neurobiology, University of Regensburg, Universitaetsstrasse 31, 93053 Regensburg; Email: virginie.rappeneau@ur.de; Phone: +49 (0) 941 943 3049.
